# Observing volcanoes with drones: studies of volcanic plume chemistry with ultralight sensor systems

**DOI:** 10.1038/s41598-022-21935-5

**Published:** 2022-10-25

**Authors:** Niklas Karbach, Nicole Bobrowski, Thorsten Hoffmann

**Affiliations:** 1 Johannes Gutenberg-University, Mainz, Germany; 2 Heidelberg University, Heidelberg, Germany; 3 Istituto Nazionale di Geofisica e Vulcanologia (INGV), Osservatorio Etneo, Catania, Italy

**Keywords:** Solid Earth sciences, Chemistry

## Abstract

The study of the chemical composition of volcanic emissions is an important method for obtaining information about volcanic systems and providing indirect and unique insights into magmatic processes. However, there is a non-negligible risk associated with sampling directly at volcanic craters or maintaining geochemical monitoring stations at such locations. Spectroscopic remote sensing methods, in turn, can measure only a few species. Here, drones offer the opportunity to bring measurement systems to the scene. Standard parameters that are commonly measured are SO_2_ and CO_2_ concentrations, as well as a number of meteorological parameters. The in-flight transmission of data by radio telemetry plays an important role, since visual localization of the volcanic plume from a distance of several kilometers is practically impossible. Until now, larger and quite cost-intensive drones have been used for this purpose, which must first be transported to the site of operation at great expense. Here, we present the development and successful deployment of a very small drone system (empty weight < 0.9 kg) for chemical characterization of volcanic plumes that can be easily transported on foot to difficult-to-access terrain and, moreover, requires only minimal flight and administrative preparations for operation as an aerial observation platform.

## Introduction

Water vapor (H_2_O), carbon dioxide (CO_2_) and sulfur dioxide (SO_2_) are the major volcanic gas components. The CO_2_/SO_2_ molar ratio is the most common parameter monitored by multi-GAS instruments today and it is a promising parameter that might contribute to anticipate eruptions in the future^1^. Ideally, this ratio is monitored continuously to analyze changes in the gas composition of a volcanic system, which occur before, during and after a volcanic eruption to minimize the risk of an unforeseen eruption^2–5^. Indeed, a change in the CO_2_/SO_2_ ratio has been observed at several volcanoes prior numerous eruptions, including Galeras^6^, Kilauea^7^, Kudriavy^8^, Villarica^9^, Aso^10^, and Etna^11,12^. An increase in the CO_2_/SO_2_ ratio is often interpreted as evidence for the injection of deep CO_2_-rich magma or magmatic fluids into the degassing region^7,11,13^. The CO_2_/SO_2_ ratio decreases when the magma moves further to shallower regions leading to a low CO_2_/SO_2_ ratio before the onset of the eruption. These changes in volcanic gases prior to the onset of volcanic activity are notable precursors to magmatic volcanic events and demonstrate the potential of a real-time gas monitoring system to anticipate volcanic eruptions. However, the practical implementation of continuous gas emission time series is challenging. Direct manual sampling is tedious, time-consuming and carries a high intrinsic risk due to a sudden onset of volcanic activity^14^. Stationary measuring stations often do not provide a representative composition of the emitted gases, especially due to changing wind directions or uncontrolled influences from different emission sources.

Airborne systems can overcome these problems and have already been used to measure the chemical composition of volcanic emissions^14–20^. They are useful for a number of reasons, including the fact that now the risk of being endangered by sudden changes in volcanic activity is substantially minimized by the researcher's increased distance to the volcano. It is also advantageous that the operator is not exposed to toxic gases during sampling or that the transport and use of respirators can be omitted. In addition, drones make it possible to reach emission sources that are otherwise difficult or impossible to access, such as fumaroles in steep, slippery terrain or older parts of the plume that are typically located in downwind areas and at higher altitudes. Also, heterogeneous gas compositions from different source regions within a crater or across a crater system are relatively easy to detect by flying over the gas emission area. Nevertheless, in remote areas, where most volcanoes are located and access by car is not possible, the drone still has to be carried on foot to the emission site, which can be quite tedious due to the heavy weight of the equipment. Currently used systems mostly belong to drone class C3 [a drone classification based mainly on weight (C0 < 250 g; C1 < 900 g; C2 < 4 kg; C3 < 25 kg)] and are therefore heavy and quite cost-intensive, especially if the drones are used regularly and the associated risk of losing the system during regular surveillance flights. Therefore, miniaturization of suitable measurement drones is a key component to reach remote or hard-to-reach volcanic regions and realize effective monitoring of volcanic activity.

The objective of this study is to demonstrate the use of an ultralight measurement drone to measure the CO_2_/SO_2_-ratio of degassing volcanos. For this purpose, a commercial C1 drone was equipped with appropriate sensors and real-time telemetry. The drone used in this study weighs only 0.9 kg, a fact that is of particular importance in practical field work, since the weight of the spare batteries comes into play here (battery weight of 0.33 kg as opposed to several kilograms in commonly used measurement drones). Of course, the maximum possible weight of the sensor and telemetry system is correspondingly lower and had to be adjusted accordingly. The developed system—little-RAVEN (little Remote-controlled Aircraft for Volcanic EmissioN analysis^21^)—was then successfully deployed during a measurement campaign on the island of Vulcano, Italy, in April 2022.

## Methodology

**Figure 1:**
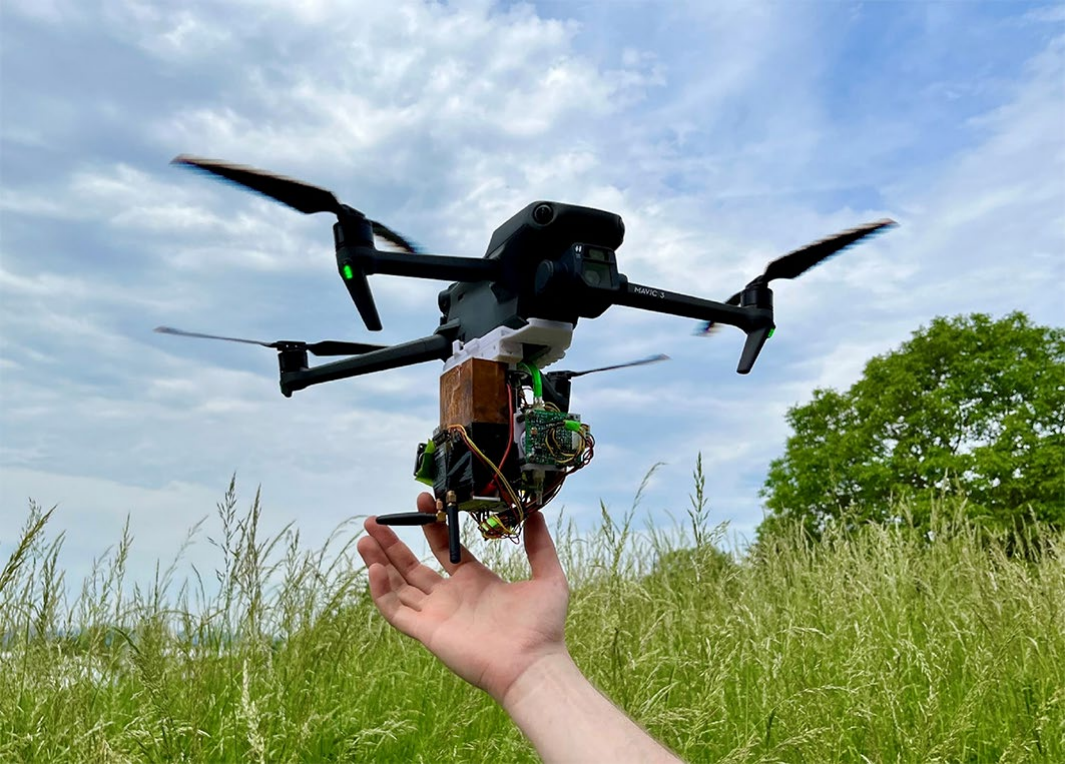
Photo of the observation drone ('little-RAVEN') during a flight test (Photo taken by T. Hoffmann).

**Figure 2:**
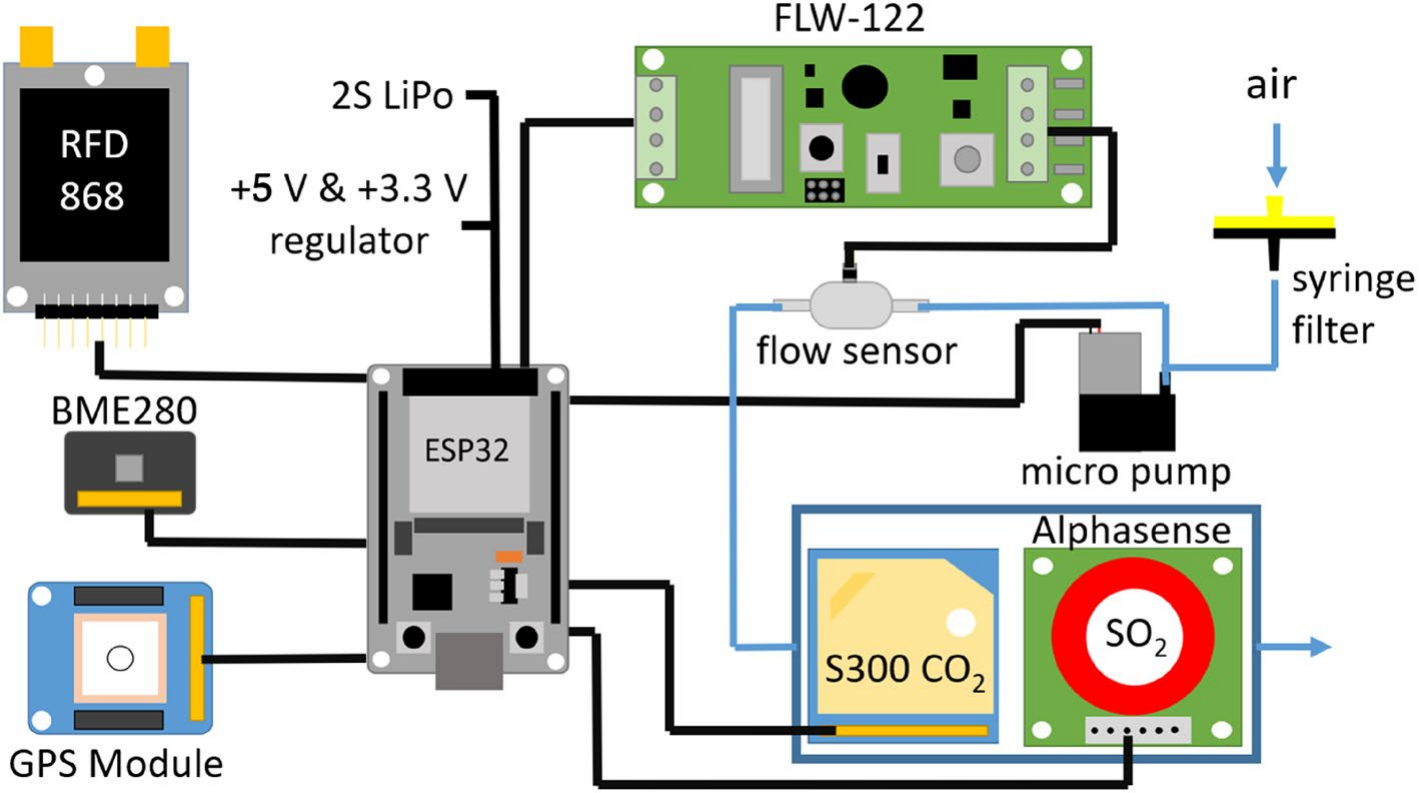
Schematic diagram of the sensor and telemetry system of little-RAVEN.

The drone used as the carrier platform is a commercial DJI Mavic 3 (total weight: 895 g; maximum service ceiling: 6000 m a.s.l.; approx. flight time: 40 min; obtained from Globe Flight, Germany). A homemade 3D-printed adapter plate (see Supplementary Information S1; weight 25.8 g) that is attached under the drone provided two universal mounting rails (modified picatinny rails) to which the payload can easily be attached. A photo of the drone with the mounting rails and the complete sensor and communication system is shown in Fig. 1. The heart of the sensor system is an ESP32 microcontroller (3.3 V; 12 bit ADC; 240 MHz; 4 MB storage) which handles the necessary communication with the various sensors, the ADC conversions, switching the sampling pump, data logging onto the internal flash memory as well as the communication with the RFD868X EU module to establish the ground connection (Fig. 2). The sensor system consists of an electrochemical SO_2_ sensor [Alphasense SO2-B4 with a corresponding “Individual Sensor Board” (ISB)], a S300 CO_2_ sensor (ELT Sensors), a pump with a flow rate of 500 mL/min, a BME280 to measure temperature, humidity and pressure and a GPS module.

The sampling rate is 0.5 Hz. The sensor system is powered via a 2S LiPo battery (1300 mAh) which is connected to a 3.3 V and a 5 V voltage regulator (LM1085) in parallel with a 4.7 µF capacitor. The total runtime exceeds 1.5 h. All components are soldered to a perfboard, which is then placed in a 3D printed box with rails to easily mount it on the adapter plate under the drone. The gas sensors (see Table 1) have an experimentally determined response time of ${\tau }_{1/e}=40 s$ for the CO_2_ sensor and ${\tau }_{1/e}=8 s$ for the SO_2_ sensor.

**Table 1:** Parts list of the sensor system with type of data transmission, function, range of the individual components, weight and approximate costs.

Module	Communication	Function	Ranges	Price	Weight
ESP32 NodeMCU (Espressif)	–	Interface for all sensors	–	~ 15€	~ 15 g
RFD868x EU (RFDesign)	UART 1	Communication with ground station	Up to 40 km	~ 323€	~ 25 g
MTK3339 GPS Module (Adafruit)	UART 2	Gets precise GPS information	–	47€	~ 9 g
BME280 (JOY-it)	I^2^C	Measures: temperature, humidity, pressure, air quality	Temp: − 40 to 85 °C Humidity: 0–100% Pressure: 0.3–1.1 kPa	17€	~ 3 g
S-300 (ELT Sensors)	I^2^C	Measures CO_2_ concentration (NDIR)	0–10,000 ppm	75€	~ 20 g
SO_2_ Sensor (Alphasense SO2-B4) with corresponding ISB	Analog output	Measures SO_2_ concentration (electrochemically)	0–15 ppm	85€	~ 50 g
Micro pump (First Sensor, T5-1HE-03-1EEB)	PWM	Controllable via PWM signal	Flow rate: 300–600 mL/min	140€	~ 14 g
FLW-122 (B + B Sensors)	Analog output	Measures the flowrate of the micro pump	Flow rate: 0–50 m/s	155€	~ 10 g
Syringe filter		Used here just as inlet filter (in future applications a potential additional sampling device		2€	~ 5 g

The software to control the microcontroller was written with the Arduino IDE (see Supplementary Information S2). For analog voltage measurements, it employs averaging of multiple measurements. All data is logged onto the internal flash memory of the microcontroller before it is sent to the ground station via the RFD868 module. This is to prevent data loss in the case of an unexpected communication error. The total weight of little-RAVEN is 1.23 kg. The total weight of little-RAVEN including the drone, backpack, two spare batteries and other necessary accessories (e.g. receiving computer at the ground) adds up to less than 3 kg at a cost of about 3.500€.

Due to the comparatively long response time of the CO_2_ sensor used, a response correction algorithm must be used to allow correct determination of the CO_2_/SO_2_ ratio. The response correction algorithm used in this work is based on a mathematical derivation of a first order step response function. To calculate the response-corrected concentration (see Eq. 1), only the slope of the measured CO_2_ function ($\frac{dc}{dt}$) and the response time of the sensor ($\uptau$) are needed. Before calculating the response-corrected concentration, the measured values are smoothed with a Savitzyk–Golay filter.

1 $${c}_{\text{corrected}}={c}_{\text{measured}}+\frac{dc}{dt}\cdot\uptau $$

For verification purposes of the CO_2_ sensor, a bigger, faster responding (${\tau }_{1/e}=4 s$), optical CO_2_ sensor, purchased from smartGAS (in the following referred to as the “fast responding sensor”), was used. The sensor outputs an analog voltage signal (0–5 V) in a range from 0 to 2000 ppm CO_2_. A detailed description of the verification procedure is given in the Supplementary Information S6. The data-processing procedure as well as the calibration procedure are described in further detail in the Supplementary Information in chapters S4 and S5.

## Results

**Figure 3:**
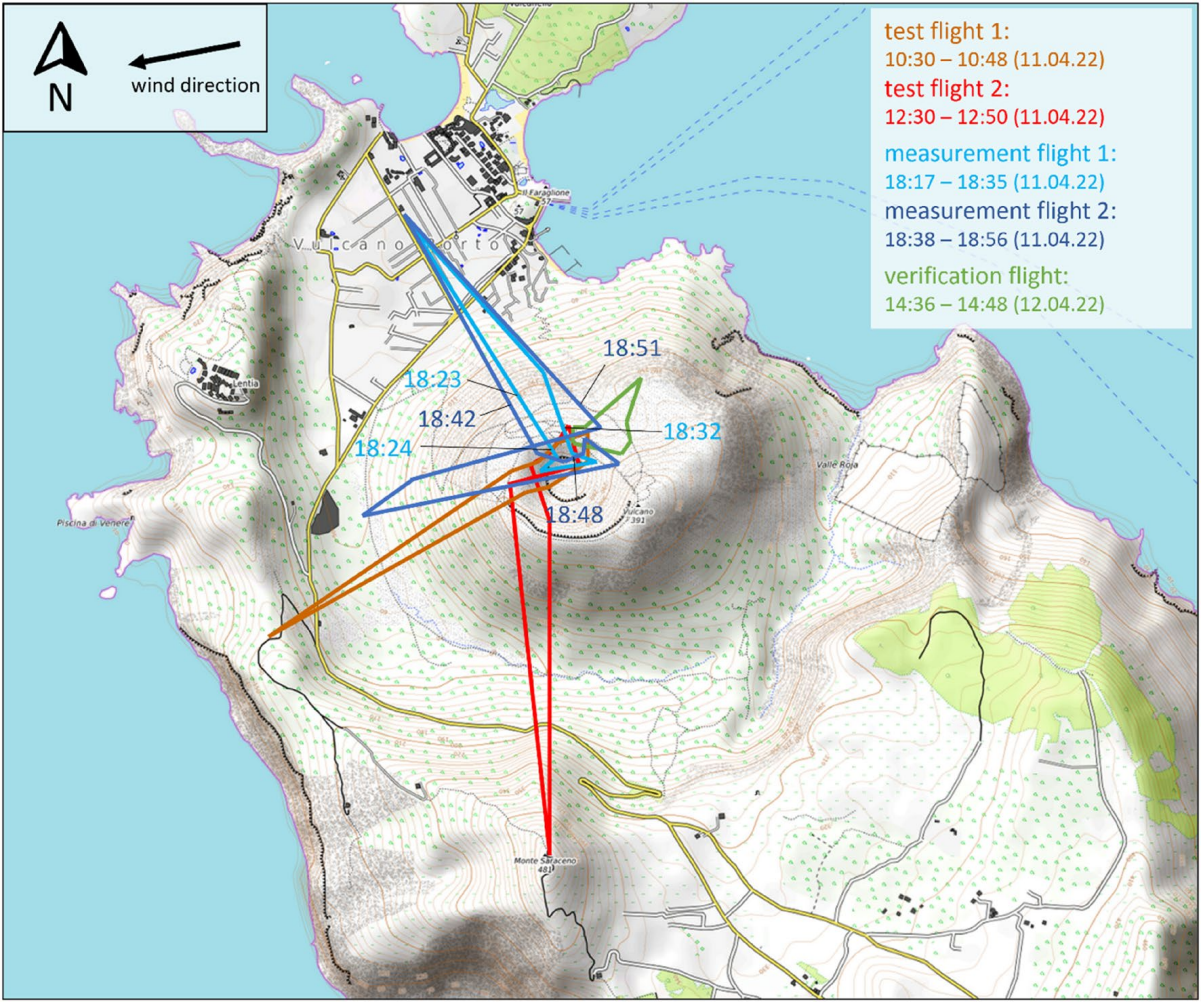
Flight paths of the first two test flights, the two measurement flights and the verification flight. The test flights and the measurement flights were conducted on the 11. April 2022. The verification flight was conducted on the 12. April 2022. (Map data: © OpenStreetMap contributors, SRTM | Map display: © OpenTopoMap (CC-BY-SA)).

The system was tested in April 2022 on the island of Vulcano, Italy. Little-RAVEN flew a total of four times, each time from a distance of ~ 1.2–1.5 km to the fumarole field. During two test flights, conducted to evaluate the flight behavior of little-RAVEN and to gain knowledge about the behavior of the telemetry system, the launch site was located on the summit of Monte Saraceno and south of Lentia, located westwards from the active La Fossa cone. During two measurement flights, the CO_2_/SO_2_ ratio of the plumes emitted by the fumarole field was measured. The takeoff and landing site was a terrace of a residential house in the village. In an additional verification flight, the sensor system was attached to a larger drone along with the fast-response optical CO_2_ sensor described above. This was done to verify the CO_2_ sensor along with the algorithm to correct the response behavior of the small sensor system. The launch point of this verification flight was located on a small ridge on the north-northeastern flank of the volcano, about 200 m from the fumarole field. The fumaroles sampled were the same fumaroles overflown during the two measurement flights the previous day. All flight paths are shown in Fig. 3.

The two measurement flights conducted on the evening of April 11, 2022, started from the village of Vulcano. To reach the measurement site (the fumarole field on the summit of the volcano, the active cone of La Fossa), little-RAVEN had to fly 1.2 km and climb from nearly sea level to ~ 270 m a.s.l.

**Figure 4:**
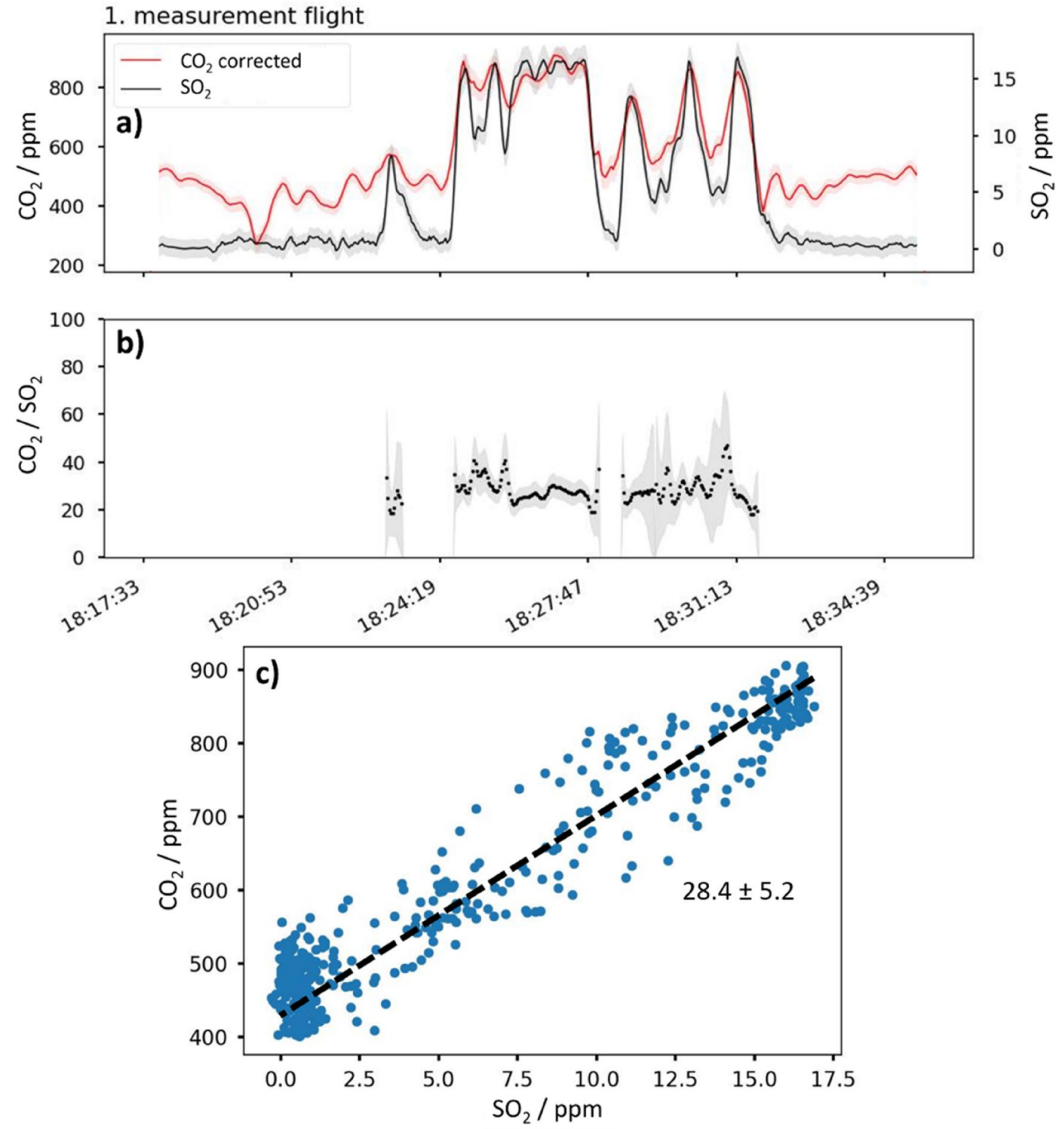
Concentration time profile (**a**) and excess-CO_2_/SO_2_-ratio (**b**) during measurement flight 1 (see Fig. 3, light blue flight path). The error was calculated according to the procedure described in the Supplementary Information S5. (**c**) CO_2_ concentration plotted against the SO_2_ concentration. All CO_2_ concentrations shown in the figures are measured with the S300 sensor and subsequently response-corrected according to the procedure described in the methodology.

During the first measurement flight (see Fig. 3, light blue flight path), the drone flew at a fairly close distance to the fumaroles, which caused the SO_2_ sensor to operate relatively close to the upper limit of its measurement range. This was avoided in the second measurement flight, however, the obtained ratios relative to the variability of CO_2_/SO_2_-ratios of different fumaroles as well as the accuracy of the measurement themselves were very similar. The averaged CO_2_/SO_2_-ratio during the first measurement flight was 28.4 ± 5.2, where the error is the standard deviation of the measured ratios and the ratio was calculated using the excess CO_2_ concentration (ambient CO_2_ concentration subtracted from the total measured CO_2_ concentration). A figure showing the time course of the measured CO_2_ and SO_2_ concentrations and their respective ratios during this first measurement flight is shown in Fig. 4a,b. Figure 4c contains a scatter plot of the measured concentrations, with the black dashed line representing the linear regression that yields the above averaged CO_2_/SO_2_ ratio. The averaged ratio between CO_2_ and SO_2_ during the second measurement flight was 32.7 ± 6.8. The two ratios calculated during the verification flight were 33.0 ± 9.3 for the data collected with little-RAVEN and 27.6 ± 9.1 for the data collected with the faster responding CO_2_ sensor. Although the volcano's activity was significantly higher during the campaign compared to previous periods, the ratios measured during the survey flights fit well with previously measured CO_2_/SO_2_-ratios at the volcano (see Refs.^22–24^). More information and the corresponding plots for the second measurement flight and the verification flight can be found in Supplementary Information, chapters S6 and S7.

## Discussion

Despite the small size and comparatively low cost of the carrier drone, the flight characteristics of the little-RAVEN system presented here are still very good in terms of flight duration, range, stability and maneuverability, and in these respects can certainly compete with the C3 drones that have so far been used as carrier drones, e.g. the DJI Matrice 210 or 300. The camera system of the drone allows easy navigation outside the direct visual range via the first-person-view mode, an indispensable advantage for flying closer to emission sources even at greater distances, e.g. for sampling degassing at summits or the flanks of active volcanoes. The camera system additionally helps to localize the plumes themselves, although real-time transmission of SO_2_ concentration remains the most reliable parameter for this purpose. The combination of sensors used here (high CO_2_ concentration range, low SO_2_ concentration range) is more suitable for characterizing CO_2_-rich volcanic emissions (such as Vulcano studied here but also Etna). In general, the expected CO_2_/SO_2_-ratios of volcanic emissions span a very wide range, usually, between 0.2 and about 10 for the high-temperature emissions from open-vent arc volcanoes^9^. It is therefore difficult to make a general recommendation on the most appropriate concentration ranges for the sensors used, although generally more sensitive sensors (higher ppb to lower ppm range) should be used to measure the ratio at a greater distance, while less sensitive sensors (ppm range) with a higher limit of quantification can be used to measure closer to the emission source. Of course, the latter measurements have the advantage that fluctuations in the background CO_2_ concentration have less impact on the determined CO_2_/SO_2_-ratios.

As can be seen on closer inspection in Fig. 4, not all effects of the slow response of the CO_2_ sensor could be removed. This is because an idealized model (first order) is used to describe the complex response behavior of the sensor. With this simplified model, the main effects of response time on the outcome of the experiments can be minimized, but not completely eliminated. Consequently, future applications of the presented system could use a faster responding, possibly actively pumped CO_2_ and SO_2_ sensor to improve the temporal response, simplify data processing, and facilitate sensor calibration. Without question, however, weight limitations must be considered when selecting the respective sensors. Again, an upper weight limit cannot be precisely specified, since increasing weight primarily affects maximum flight time (but also flight stability). However, an additional weight of > 330 g is not advisable based on the test flights presented here. The results presented in this paper further show that the use of a slow response CO_2_ sensor, which was used here especially because of weight reasons, in combination with a computational correction of the response provides the same ratios within their respective error as recorded with a fast response sensor. This conclusion could be confirmed in laboratory experiments (see Supplementary Information, chapter S3). In these experiments, different CO_2_ concentrations were generated as short concentration peaks in a test gas atmosphere and measured with the slow-response CO_2_ sensor used in the drone. The results of this experiment show that the differences between the appropriately corrected values measured with the slow-response sensor and the concentration measured with the fast-response sensor are negligible.

## Conclusions

This paper demonstrates that miniaturized sensor systems carried by small, lightweight drones are a viable option for gas measurements in volcanic emissions. Based on a commercial 900 g drone equipped with suitable lightweight sensors, SO_2_ and CO_2_ concentrations were determined as well as a number of environmental parameters such as pressure, temperature, and relative humidity during a measurement campaign at Vulcano, Italy. The averaged excess-CO_2_/SO_2_-ratio that was measured is 31.4 ± 7.1. Beyond a direct visual contact, real-time in-flight data transmission via radio is an essential requirement for the remote measurements performed, as both orography and current meteorology make it difficult to locate the volcanic plume from several kilometers away. With the presented measurement system, new measurement sites at volcanoes, which are otherwise difficult to access, can be explored. The system also enables higher measurement frequencies for monitoring volcanic activity through gas measurements. The C1 drone used here has recently been formally approved for a C1 certification in the European Economic Area (i.e. EU plus Norway, Iceland and Liechtenstein), under the new European Drone Regulation (https://www.easa.europa.eu/domains/civil-drones). This means that the operator of the drone in the so-called open category can now just fly with the A1 license (EU 'certificate of competency' in the A1 subcategory) instead of the more regulated and more difficult to obtain A2 license. With the C1-certified drone used here, flights in more regions and environments are feasible without the need for additional administrative procedures and special permits, as is the case with larger drones without C1 certification. In addition, the easy-to-operate and safe systems allow monitoring tasks at comparatively low costs, which is an argument not to be neglected in volcano monitoring for geological/governmental institutions in economically weak regions.

## Supplementary Information

Supplementary Information.

## Data Availability

All raw-data is available at: https://github.com/NKa1409/little-RAVEN_Vulcano2022.
