# Supplementary material for: Observing volcanoes with drones: studies of volcanic plume chemistry with ultralight sensor systems
Source: Sci Rep. 2022 Oct 25;12:17890. doi: 10.1038/s41598-022-21935-5 (PMC9596470; doi:10.1038/s41598-022-21935-5)
Supplement: Supplementary file 1 — Supplementary Information. [file 41598_2022_21935_MOESM1_ESM.docx]

Observing volcanoes with drones: studies of volcanic plume chemistry with ultralight sensor systems

Niklas Karbach^1^, Nicole Bobrowski^2,3^, Thorsten Hoffmann*^1^

1 Johannes Gutenberg-University Mainz, Germany

2 Heidelberg University, Germany

3 Istituto Nazionale di Geofisica e Vulcanologia (INGV) Catania, Italy

# Adapterplate

The tailor-made adapterplate for the Mavic 3 drone, provides two universal mounting rails (customized picatinny rails) to quickly attach and interchange the sensorsystem. The adapterplate is easily attachable and detachable from the drone via a click-mechanism. The sensorsystem itself just slides onto the rails and holds in place by friction.

The adapterplate was designed with Fusion 360 and then 3D printed with PLA (polylactic acid). The total weight is 25.8 g.

# Microcontroller code

The microcontroller handles all the necessary communication, computation and storage requirements of the various sensors and modules which are used on the sensorsystem itself. In the following, a short overview over the individual functions is given.

For debugging and testing purposes three serial interfaces are implemented on the microcontroller. The serial interfaces are the “normal” USB serial interface, a Bluetooth serial interface and the serial interface through which the RFD868 module communicates with the ground station. In the normal application, all information between the user and the microcontroller is exchanged via the RFD868 module. This includes sending the data to the ground station, but also changing the PWM duty cycle of the sampling pump and managing the logfile, which is stored on the microcontroller, from the ground station.

The SO_2_ sensor and the flowmeter provide an analog output, which is read by the microcontroller at the corresponding pin. The measurement is done 10 times and the average is calculated to reduce noise.

The CO_2_ sensor and the BME280 communicate via I^2^C. For the CO_2_ sensor, the concentration is received directly in ppm and for the BME280 module the temperature, humidity and pressure are received in °C, %rH and Pa respectively. The communication of the CO_2_ sensor is controlled by the “s300i2c.h” library (by *mhorimoto*). The communication of the BME280 module is controlled by the “Adafruit_BME280.h” library (by *Adafruit*).

The GPS module and the RFD868 module communicate with the microcontroller via a serial interface. The communication with the GPS module is controlled by the “Adafruit_GPS.h” library (by *Adafruit*). The RFD868 module is directly controlled by the microcontroller, as it only functions as a relay to send serial data.

The pump is controlled by a transistor, which is in turn controlled by a PWM signal provided by a pin of the microcontroller. The PWM duty cycle that controls the pump can be adjusted via all three serial interfaces (USB serial, Bluetooth serial and RFD868 serial).

All data that is acquired by the microcontroller is not only send to the ground station via the RFD868, but also stored on the internal flash memory of the microcontroller itself. This prevents data loss in the case of an unexpected communication error. The filesystem is handled by the “LITTLEFS.h” library (by *lorol*).

// LittleFS initialization
#include <Arduino.h>
#include <LITTLEFS.h>
bool Status_File_System = false;
bool initialize_littlefs_format_file_system() {
 if (!LITTLEFS.begin(false)) {
 Serial.println("LITTLEFS mount failed");
 Serial.println("No filesystem found; formatting...");
 if (!LITTLEFS.begin(true)) {
 Serial.println("LITTLEFS mount failed");
 Serial.println("Formatting not possible");
 return false;
 } else {
 Serial.println("Formatting successful");
 Serial.println("Information on the filesystem:");
 Serial.printf("- Bytes total: %ld\n", LITTLEFS.totalBytes());
 Serial.printf("- Bytes used: %ld\n\n", LITTLEFS.usedBytes());
 return true;
 }
 }
 Serial.println("Information on the filesystem:");
 Serial.printf("- Bytes total: %ld\n", LITTLEFS.totalBytes());
 Serial.printf("- Bytes used: %ld\n\n", LITTLEFS.usedBytes());
 return true;
}
bool write_string_to_file(String filename, String string) {
 File file = LITTLEFS.open(filename, "a");
 if (!file) {
 Serial.println("There was an error opening the file for appending");
 return false;
 }
 if (file.print(string)) {
 file.close();
 return true;
 } else {
 Serial.println("File write failed");
 file.close();
 return false;
 }
 file.close();
 return false;
}
bool read_file_and_print_to_serial(String filename) {
 File file = LITTLEFS.open(filename, "r");
 if (!file) {
 Serial.println("There was an error opening the file for reading");
 return false;
 }
 while (file.available()) {
 Serial.write(file.read());
 }
 file.close();
 return true;
}
bool delete_file(String filename) {
 Serial.print("Delete file: ");
 Serial.print(filename);
 Serial.print(" ");
 if (LITTLEFS.remove(filename)) {
 Serial.println("- File deleted");
 return true;
 } else {
 Serial.println("- File could not be deleted");
 return false;
 }
 return false;
}


// Serial communication implementation
char StringInputSpeicher[500];
void InitialiseSerial(int BaudRate) {
 Serial.begin(BaudRate); // Start serial monitor at 115200 (ESP32)
 delay(500);
 Serial.println("Serial connection established.");
 if (readSerialTo(StringInputSpeicher)) {
 Serial.println("Old data that was stored in buffer:");
 Serial.println(StringInputSpeicher);
 }
}
bool readSerialTo(char serialSpeicher[]) {
 const int SERIAL_BUFFER_SIZE = 500;
 static char serialBuffer[SERIAL_BUFFER_SIZE];
 static byte index;
 while (Serial.available()) {
 char c = Serial.read();

 if (c == '\n' && index > 0) {
 serialBuffer[index] = '\0';
 index = 0;
 strcpy(serialSpeicher, serialBuffer);
 return true;
 }
 else if (c >= 32 && index < SERIAL_BUFFER_SIZE - 1) {
 serialBuffer[index++] = c;
 }
 }
 return false;
}


// Bluetooth Serial communication implementation
#include "BluetoothSerial.h"
bool BTSwitchedOn = false;
BluetoothSerial ESP_BT; //Object for Bluetooth
char BluetoothInputSpeicher[500];
bool readBTSerialTo(char BTInputBuffer[]) {
 const int BTSERIAL_BUFFER_SIZE = 500;
 static char BTserialBuffer[BTSERIAL_BUFFER_SIZE];
 static byte BTindex;
 while (ESP_BT.available()) {
 char BTc = ESP_BT.read();
 if (BTc == '\n' && BTindex > 0) {
 BTserialBuffer[BTindex] = '\0';
 BTindex = 0;
 strcpy(BTInputBuffer, BTserialBuffer);
 return true;
 }
 else if (BTc >= 32 && BTindex < BTSERIAL_BUFFER_SIZE - 1) {
 BTserialBuffer[BTindex++] = BTc;
 }
 }
 return false;
}
void InitialiseBluetooth() {
 if (!ESP_BT.begin("ESP32_BluetoothSensor")) {
 Serial.println("An error occurred initializing Bluetooth");
 } else {
 Serial.println("Bluetooth initialized");
 }
 if (readBTSerialTo(BluetoothInputSpeicher)) {
 Serial.println("Old data that was stored in the buffer:");
 Serial.println(BluetoothInputSpeicher);
 }
 BTSwitchedOn = true;
}


// RFD communication implementation
#include <HardwareSerial.h>
HardwareSerial SerialRFD(1); // Hardware serial object = RFD object
char RFDStringInputSpeicher[500];
void InitialiseRFDSerial() {
 SerialRFD.begin(57600, SERIAL_8N1, 13, 14);
 if(SerialRFD){
 Serial.println("SerialRFD successfully set up");
 }

 if (readSerialRFDTo(RFDStringInputSpeicher)) {
 Serial.println("Old data that was stored in the buffer:");
 Serial.println(RFDStringInputSpeicher);
 }
}
bool readSerialRFDTo(char serialSpeicher[]) {
 const int SERIAL_BUFFER_SIZE = 500;
 static char serialBuffer[SERIAL_BUFFER_SIZE];
 static byte index;
 while (SerialRFD.available()) {
 char c = SerialRFD.read();

 if (c == '\n' && index > 0) {
 serialBuffer[index] = '\0';
 index = 0;
 strcpy(serialSpeicher, serialBuffer);
 return true;
 }
 else if (c >= 32 && index < SERIAL_BUFFER_SIZE - 1) {
 serialBuffer[index++] = c;
 }
 }
 return false;
}


// read a SI voltage from a pin
float readSIVoltageFromPin(int volt_pin, int anzahl_spannungs_messung, int x_bit_adc, float max_voltage) {
 float si_voltage = 0;
 long long voltage = 0;
 if (anzahl_spannungs_messung >= 300000) {
 anzahl_spannungs_messung = 300000;
 }
 if (anzahl_spannungs_messung <= 0) {
 return 0;
 }
 delay(3);

 for (int i = 0; i < anzahl_spannungs_messung; i++) {
 voltage = voltage + analogRead(volt_pin);
 }
 int total_adc_res = int(pow(2, x_bit_adc));
 si_voltage = float(voltage) / float(anzahl_spannungs_messung);
 si_voltage = (si_voltage * max_voltage) / total_adc_res;
 return si_voltage;
}


// BME implementation
// BME communication via I2C Pins
#include <Wire.h>
#include <SPI.h>
#include <Adafruit_Sensor.h>
#include <Adafruit_BME280.h>
Adafruit_BME280 bme; // BME object
float Temperature = 0;
float Pressure = 0;
float Humidity = 0;
bool StatusBMESensor = false;
bool initialize_bme_sensor() {
 StatusBMESensor = bme.begin(0x77);
 if (!StatusBMESensor) {
 Serial.println("Could not find a valid BME280 sensor!");
 StatusBMESensor = false;
 return false;
 }
 Serial.println("Initialization of BME Sensor done.");
 StatusBMESensor = true;
 return true;
}


// GPS implementation
// Communication via serial connection
#include <HardwareSerial.h>
HardwareSerial GPSSerial(2);
#include <Adafruit_GPS.h>
Adafruit_GPS GPS(&GPSSerial); // GPS object
char c;
bool status_GPS_module = false;
int GPS_timeout = 4000;
bool clearGPS() { // Delete previously acquired data from GPS
 int start_millis = millis();
 while (!GPS.newNMEAreceived()) {
 c = GPS.read();
 if (((millis() - start_millis) >= GPS_timeout) or (millis() < start_millis)) {
 status_GPS_module = false;
 break;
 }
 }
 GPS.parse(GPS.lastNMEA());

 start_millis = millis();
 while (!GPS.newNMEAreceived()) {
 c = GPS.read();
 if (((millis() - start_millis) >= GPS_timeout) or (millis() < start_millis)) {
 status_GPS_module = false;
 break;
 }
 }
 GPS.parse(GPS.lastNMEA());

 start_millis = millis();
 while (!GPS.newNMEAreceived()) {
 c = GPS.read();
 if (((millis() - start_millis) >= GPS_timeout) or (millis() < start_millis)) {
 status_GPS_module = false;
 return false;
 break;
 }
 }
 GPS.parse(GPS.lastNMEA());
 return true;
}
bool initialize_GPS() { // Setup of GPS Module
 GPS.begin(9600);
 GPS.sendCommand(PMTK_SET_NMEA_OUTPUT_RMCGGA);
 GPS.sendCommand(PMTK_SET_NMEA_UPDATE_1HZ);
 delay(100);
 status_GPS_module = clearGPS();
 if (status_GPS_module) {
 Serial.println("GPS connection established!");
 return true;
 }
 else {
 Serial.println("GPS initialization failed!");
 return false;
 }
 return true;
}


// S300 Sensor
int co2 = 0;
#include "s300i2c.h"
#include <Wire.h>
S300I2C s3(Wire); // S300 object
bool status_s300 = false;
bool initialize_s300() {
 Wire.begin();
 s3.begin(S300I2C_ADDR);
 delay(10000);
 s3.wakeup();
 s3.end_mcdl();
 s3.end_acdl();
 Serial.println("S300 CO2 Initialized");
 return true;
}
int get_co2() {
 co2 = s3.getCO2ppm();
 return co2;
}


int pump_pwm = 0;
String filename = "/datalogger.txt";
void setup() {
 // put your setup code here, to run once:
 InitialiseSerial(115200);
 InitialiseRFDSerial();
 InitialiseBluetooth();


 pinMode(12, INPUT);
 pinMode(26, INPUT);
 pinMode(34, INPUT);


 Status_File_System = initialize_littlefs_format_file_system();
 if (Status_File_System) {
 Serial.println("File system initialized");
 }


 StatusBMESensor = initialize_bme_sensor();


 status_GPS_module = initialize_GPS();


 status_s300 = initialize_s300();


 // PWM Pin implementation (pump)
 pinMode(19, OUTPUT);
 ledcSetup(0, 200, 8);
 ledcAttachPin(19, 0);


 Serial.println("Setup finished");
}


void loop() {
 // put your main code here, to run repeatedly:
 String write_to_file_string = "";
 write_to_file_string += String(millis());
 write_to_file_string += ",";


 // Try to read serial input and execute command
 if (readSerialTo(StringInputSpeicher)) {
 Serial.println("Serial input recieved!");
 Serial.println(StringInputSpeicher);
 if ((strcmp(StringInputSpeicher, "help")) == 0) {
 Serial.print("Print Datalogging file to serial terminal: ");
 Serial.println("'print data'");
 Serial.print("Delete Datalogging file: ");
 Serial.println("'delete data'");
 Serial.print("Switch pump on: ");
 Serial.println("'pump_on'");
 Serial.print("Switch pump off: ");
 Serial.println("'pump_off'");
 Serial.print("Switch pump 10/255: ");
 Serial.println("'10'");
 Serial.print("Switch pump 20/255: ");
 Serial.println("'20'");
 Serial.print("Switch pump 30/255: ");
 Serial.println("'30'");
 Serial.println("...");
 Serial.println();
 }
 else if ((strcmp(StringInputSpeicher, "print data")) == 0) {
 read_file_and_print_to_serial(filename);
 }
 else if ((strcmp(StringInputSpeicher, "delete data")) == 0) {
 delete_file(filename);
 }
 else if ((strcmp(StringInputSpeicher, "pump_on")) == 0) {
 digitalWrite(26, HIGH);
 ledcWrite(0, 254);
 pump_pwm = 254;
 }
 else if ((strcmp(StringInputSpeicher, "pump_off")) == 0) {
 digitalWrite(26, LOW);
 ledcWrite(0, 0);
 pump_pwm = 0;
 }
 else if ((strcmp(StringInputSpeicher, "0")) == 0) {
 ledcWrite(0, 0);
 pump_pwm = 0;
 }
 else if ((strcmp(StringInputSpeicher, "10")) == 0) {
 ledcWrite(0, 10);
 pump_pwm = 10;
 }
 else if ((strcmp(StringInputSpeicher, "20")) == 0) {
 ledcWrite(0, 20);
 pump_pwm = 20;
 }
 else if ((strcmp(StringInputSpeicher, "30")) == 0) {
 ledcWrite(0, 30);
 pump_pwm = 30;
 }
 else if ((strcmp(StringInputSpeicher, "40")) == 0) {
 ledcWrite(0, 40);
 pump_pwm = 40;
 }
 else if ((strcmp(StringInputSpeicher, "50")) == 0) {
 ledcWrite(0, 50);
 pump_pwm = 50;
 }
 else if ((strcmp(StringInputSpeicher, "60")) == 0) {
 ledcWrite(0, 60);
 pump_pwm = 60;
 }
 else if ((strcmp(StringInputSpeicher, "70")) == 0) {
 ledcWrite(0, 70);
 pump_pwm = 70;
 }
 else if ((strcmp(StringInputSpeicher, "80")) == 0) {
 ledcWrite(0, 80);
 pump_pwm = 80;
 }
 else if ((strcmp(StringInputSpeicher, "90")) == 0) {
 ledcWrite(0, 90);
 pump_pwm = 90;
 }
 else if ((strcmp(StringInputSpeicher, "100")) == 0) {
 ledcWrite(0, 100);
 pump_pwm = 100;
 }
 else if ((strcmp(StringInputSpeicher, "110")) == 0) {
 ledcWrite(0, 110);
 pump_pwm = 110;
 }
 else if ((strcmp(StringInputSpeicher, "120")) == 0) {
 ledcWrite(0, 120);
 pump_pwm = 120;
 }
 else if ((strcmp(StringInputSpeicher, "130")) == 0) {
 ledcWrite(0, 130);
 pump_pwm = 130;
 }
 else if ((strcmp(StringInputSpeicher, "140")) == 0) {
 ledcWrite(0, 140);
 pump_pwm = 140;
 }
 else if ((strcmp(StringInputSpeicher, "150")) == 0) {
 ledcWrite(0, 150);
 pump_pwm = 150;
 }
 else if ((strcmp(StringInputSpeicher, "160")) == 0) {
 ledcWrite(0, 160);
 pump_pwm = 160;
 }
 else if ((strcmp(StringInputSpeicher, "170")) == 0) {
 ledcWrite(0, 170);
 pump_pwm = 170;
 }
 else if ((strcmp(StringInputSpeicher, "180")) == 0) {
 ledcWrite(0, 180);
 pump_pwm = 180;
 }
 else if ((strcmp(StringInputSpeicher, "190")) == 0) {
 ledcWrite(0, 190);
 pump_pwm = 190;
 }
 else if ((strcmp(StringInputSpeicher, "200")) == 0) {
 ledcWrite(0, 200);
 pump_pwm = 200;
 }
 else if ((strcmp(StringInputSpeicher, "210")) == 0) {
 ledcWrite(0, 210);
 pump_pwm = 210;
 }
 else if ((strcmp(StringInputSpeicher, "220")) == 0) {
 ledcWrite(0, 220);
 pump_pwm = 220;
 }
 else if ((strcmp(StringInputSpeicher, "230")) == 0) {
 ledcWrite(0, 230);
 pump_pwm = 230;
 }
 else if ((strcmp(StringInputSpeicher, "240")) == 0) {
 ledcWrite(0, 240);
 pump_pwm = 240;
 }
 else if ((strcmp(StringInputSpeicher, "250")) == 0) {
 ledcWrite(0, 250);
 pump_pwm = 250;
 }

 }

 // Try to read Bluetooth serial input and execute command
 if (readBTSerialTo(BluetoothInputSpeicher)) {
 ESP_BT.println("Serial input recieved!");
 ESP_BT.println(BluetoothInputSpeicher);
 if ((strcmp(BluetoothInputSpeicher, "help")) == 0) {
 ESP_BT.print("Print Datalogging file to serial terminal: ");
 ESP_BT.println("'print data'");
 ESP_BT.print("Delete Datalogging file: ");
 ESP_BT.println("'delete data'");
 ESP_BT.print("Switch pump on: ");
 ESP_BT.println("'pump_on'");
 ESP_BT.print("Switch pump off: ");
 ESP_BT.println("'pump_off'");
 ESP_BT.print("Switch pump 10/255: ");
 ESP_BT.println("'10'");
 ESP_BT.print("Switch pump 20/255: ");
 ESP_BT.println("'20'");
 ESP_BT.print("Switch pump 30/255: ");
 ESP_BT.println("'30'");
 ESP_BT.println("...");
 ESP_BT.println();
 }
 else if ((strcmp(BluetoothInputSpeicher, "print data")) == 0) {
 read_file_and_print_to_serial(filename);
 }
 else if ((strcmp(BluetoothInputSpeicher, "delete data")) == 0) {
 delete_file(filename);
 }
 else if ((strcmp(BluetoothInputSpeicher, "pump_on")) == 0) {
 digitalWrite(26, HIGH);
 ledcWrite(0, 254);
 pump_pwm = 254;
 }
 else if ((strcmp(BluetoothInputSpeicher, "pump_off")) == 0) {
 digitalWrite(26, LOW);
 ledcWrite(0, 0);
 pump_pwm = 0;
 }
 else if ((strcmp(BluetoothInputSpeicher, "0")) == 0) {
 ledcWrite(0, 0);
 pump_pwm = 0;
 }
 else if ((strcmp(BluetoothInputSpeicher, "10")) == 0) {
 ledcWrite(0, 10);
 pump_pwm = 10;
 }
 else if ((strcmp(BluetoothInputSpeicher, "20")) == 0) {
 ledcWrite(0, 20);
 pump_pwm = 20;
 }
 else if ((strcmp(BluetoothInputSpeicher, "30")) == 0) {
 ledcWrite(0, 30);
 pump_pwm = 30;
 }
 else if ((strcmp(BluetoothInputSpeicher, "40")) == 0) {
 ledcWrite(0, 40);
 pump_pwm = 40;
 }
 else if ((strcmp(BluetoothInputSpeicher, "50")) == 0) {
 ledcWrite(0, 50);
 pump_pwm = 50;
 }
 else if ((strcmp(BluetoothInputSpeicher, "60")) == 0) {
 ledcWrite(0, 60);
 pump_pwm = 60;
 }
 else if ((strcmp(BluetoothInputSpeicher, "70")) == 0) {
 ledcWrite(0, 70);
 pump_pwm = 70;
 }
 else if ((strcmp(BluetoothInputSpeicher, "80")) == 0) {
 ledcWrite(0, 80);
 pump_pwm = 80;
 }
 else if ((strcmp(BluetoothInputSpeicher, "90")) == 0) {
 ledcWrite(0, 90);
 pump_pwm = 90;
 }
 else if ((strcmp(BluetoothInputSpeicher, "100")) == 0) {
 ledcWrite(0, 100);
 pump_pwm = 100;
 }
 else if ((strcmp(BluetoothInputSpeicher, "110")) == 0) {
 ledcWrite(0, 110);
 pump_pwm = 110;
 }
 else if ((strcmp(BluetoothInputSpeicher, "120")) == 0) {
 ledcWrite(0, 120);
 pump_pwm = 120;
 }
 else if ((strcmp(BluetoothInputSpeicher, "130")) == 0) {
 ledcWrite(0, 130);
 pump_pwm = 130;
 }
 else if ((strcmp(BluetoothInputSpeicher, "140")) == 0) {
 ledcWrite(0, 140);
 pump_pwm = 140;
 }
 else if ((strcmp(BluetoothInputSpeicher, "150")) == 0) {
 ledcWrite(0, 150);
 pump_pwm = 150;
 }
 else if ((strcmp(BluetoothInputSpeicher, "160")) == 0) {
 ledcWrite(0, 160);
 pump_pwm = 160;
 }
 else if ((strcmp(BluetoothInputSpeicher, "170")) == 0) {
 ledcWrite(0, 170);
 pump_pwm = 170;
 }
 else if ((strcmp(BluetoothInputSpeicher, "180")) == 0) {
 ledcWrite(0, 180);
 pump_pwm = 180;
 }
 else if ((strcmp(BluetoothInputSpeicher, "190")) == 0) {
 ledcWrite(0, 190);
 pump_pwm = 190;
 }
 else if ((strcmp(BluetoothInputSpeicher, "200")) == 0) {
 ledcWrite(0, 200);
 pump_pwm = 200;
 }
 else if ((strcmp(BluetoothInputSpeicher, "210")) == 0) {
 ledcWrite(0, 210);
 pump_pwm = 210;
 }
 else if ((strcmp(BluetoothInputSpeicher, "220")) == 0) {
 ledcWrite(0, 220);
 pump_pwm = 220;
 }
 else if ((strcmp(BluetoothInputSpeicher, "230")) == 0) {
 ledcWrite(0, 230);
 pump_pwm = 230;
 }
 else if ((strcmp(BluetoothInputSpeicher, "240")) == 0) {
 ledcWrite(0, 240);
 pump_pwm = 240;
 }
 else if ((strcmp(BluetoothInputSpeicher, "250")) == 0) {
 ledcWrite(0, 250);
 pump_pwm = 250;
 }

 }

 // Try to read RFD serial input and execute command
 if (readSerialRFDTo(RFDStringInputSpeicher)) {
 SerialRFD.println("Serial input recieved!");
 SerialRFD.println(RFDStringInputSpeicher);
 if ((strcmp(RFDStringInputSpeicher, "help")) == 0) {
 SerialRFD.print("Print Datalogging file to serial terminal: ");
 SerialRFD.println("'print data'");
 SerialRFD.print("Delete Datalogging file: ");
 SerialRFD.println("'delete data'");
 SerialRFD.print("Switch pump on: ");
 SerialRFD.println("'pump_on'");
 SerialRFD.print("Switch pump off: ");
 SerialRFD.println("'pump_off'");
 SerialRFD.print("Switch pump 10/255: ");
 SerialRFD.println("'10'");
 SerialRFD.print("Switch pump 20/255: ");
 SerialRFD.println("'20'");
 SerialRFD.print("Switch pump 30/255: ");
 SerialRFD.println("'30'");
 SerialRFD.println("...");
 SerialRFD.println();
 }
 else if ((strcmp(RFDStringInputSpeicher, "print data")) == 0) {
 read_file_and_print_to_serial(filename);
 }
 else if ((strcmp(RFDStringInputSpeicher, "delete data")) == 0) {
 delete_file(filename);
 }
 else if ((strcmp(RFDStringInputSpeicher, "pump_on")) == 0) {
 digitalWrite(26, HIGH);
 ledcWrite(0, 254);
 pump_pwm = 254;
 }
 else if ((strcmp(RFDStringInputSpeicher, "pump_off")) == 0) {
 digitalWrite(26, LOW);
 ledcWrite(0, 0);
 pump_pwm = 0;
 }
 else if ((strcmp(RFDStringInputSpeicher, "0")) == 0) {
 ledcWrite(0, 0);
 pump_pwm = 0;
 }
 else if ((strcmp(RFDStringInputSpeicher, "10")) == 0) {
 ledcWrite(0, 10);
 pump_pwm = 10;
 }
 else if ((strcmp(RFDStringInputSpeicher, "20")) == 0) {
 ledcWrite(0, 20);
 pump_pwm = 20;
 }
 else if ((strcmp(RFDStringInputSpeicher, "30")) == 0) {
 ledcWrite(0, 30);
 pump_pwm = 30;
 }
 else if ((strcmp(RFDStringInputSpeicher, "40")) == 0) {
 ledcWrite(0, 40);
 pump_pwm = 40;
 }
 else if ((strcmp(RFDStringInputSpeicher, "50")) == 0) {
 ledcWrite(0, 50);
 pump_pwm = 50;
 }
 else if ((strcmp(RFDStringInputSpeicher, "60")) == 0) {
 ledcWrite(0, 60);
 pump_pwm = 60;
 }
 else if ((strcmp(RFDStringInputSpeicher, "70")) == 0) {
 ledcWrite(0, 70);
 pump_pwm = 70;
 }
 else if ((strcmp(RFDStringInputSpeicher, "80")) == 0) {
 ledcWrite(0, 80);
 pump_pwm = 80;
 }
 else if ((strcmp(RFDStringInputSpeicher, "90")) == 0) {
 ledcWrite(0, 90);
 pump_pwm = 90;
 }
 else if ((strcmp(RFDStringInputSpeicher, "100")) == 0) {
 ledcWrite(0, 100);
 pump_pwm = 100;
 }
 else if ((strcmp(RFDStringInputSpeicher, "110")) == 0) {
 ledcWrite(0, 110);
 pump_pwm = 110;
 }
 else if ((strcmp(RFDStringInputSpeicher, "120")) == 0) {
 ledcWrite(0, 120);
 pump_pwm = 120;
 }
 else if ((strcmp(RFDStringInputSpeicher, "130")) == 0) {
 ledcWrite(0, 130);
 pump_pwm = 130;
 }
 else if ((strcmp(RFDStringInputSpeicher, "140")) == 0) {
 ledcWrite(0, 140);
 pump_pwm = 140;
 }
 else if ((strcmp(RFDStringInputSpeicher, "150")) == 0) {
 ledcWrite(0, 150);
 pump_pwm = 150;
 }
 else if ((strcmp(RFDStringInputSpeicher, "160")) == 0) {
 ledcWrite(0, 160);
 pump_pwm = 160;
 }
 else if ((strcmp(RFDStringInputSpeicher, "170")) == 0) {
 ledcWrite(0, 170);
 pump_pwm = 170;
 }
 else if ((strcmp(RFDStringInputSpeicher, "180")) == 0) {
 ledcWrite(0, 180);
 pump_pwm = 180;
 }
 else if ((strcmp(RFDStringInputSpeicher, "190")) == 0) {
 ledcWrite(0, 190);
 pump_pwm = 190;
 }
 else if ((strcmp(RFDStringInputSpeicher, "200")) == 0) {
 ledcWrite(0, 200);
 pump_pwm = 200;
 }
 else if ((strcmp(RFDStringInputSpeicher, "210")) == 0) {
 ledcWrite(0, 210);
 pump_pwm = 210;
 }
 else if ((strcmp(RFDStringInputSpeicher, "220")) == 0) {
 ledcWrite(0, 220);
 pump_pwm = 220;
 }
 else if ((strcmp(RFDStringInputSpeicher, "230")) == 0) {
 ledcWrite(0, 230);
 pump_pwm = 230;
 }
 else if ((strcmp(RFDStringInputSpeicher, "240")) == 0) {
 ledcWrite(0, 240);
 pump_pwm = 240;
 }
 else if ((strcmp(RFDStringInputSpeicher, "250")) == 0) {
 ledcWrite(0, 250);
 pump_pwm = 250;
 }
 }


 // Get data from BME Sensor
 if (StatusBMESensor) {
 Temperature = bme.readTemperature();
 Pressure = bme.readPressure();
 Pressure = Pressure / 100;
 Humidity = bme.readHumidity();
 write_to_file_string += String(Temperature);
 write_to_file_string += ",";
 write_to_file_string += String(Pressure);
 write_to_file_string += ",";
 write_to_file_string += String(Humidity);
 write_to_file_string += ",";
 }
 else {
 Serial.println("No BME sensor available!!");
 ESP_BT.println("No BME sensor available!!");
 SerialRFD.println("No BME sensor available!!");
 StatusBMESensor = initialize_bme_sensor();
 write_to_file_string += "No BME Sensor available,,,";
 }


 // Get analog voltages from pins
 // flow meter
 float SI_voltage_pin_12 = readSIVoltageFromPin(12, 10, 12, 3.3);
 if (SI_voltage_pin_12 >= 3.7) {
 Serial.print("Problem with ADC on Pin 12; measured voltage=");
 Serial.println(SI_voltage_pin_12);
 ESP_BT.print("Problem with ADC on Pin 12; measured voltage=");
 ESP_BT.println(SI_voltage_pin_12);
 SerialRFD.print("Problem with ADC on Pin 12; measured voltage=");
 SerialRFD.println(SI_voltage_pin_12);
 }
 // SO2
 float SI_voltage_pin_26= readSIVoltageFromPin(26, 10, 12, 3.3);
 if (SI_voltage_pin_26 >= 3.7) {
 Serial.print("Problem with ADC on Pin 26; measured voltage=");
 Serial.println(SI_voltage_pin_26);
 ESP_BT.print("Problem with ADC on Pin 26; measured voltage=");
 ESP_BT.println(SI_voltage_pin_26);
 SerialRFD.print("Problem with ADC on Pin 26; measured voltage=");
 SerialRFD.println(SI_voltage_pin_26);
 }
 // NC
 float SI_voltage_pin_34 = readSIVoltageFromPin(34, 10, 12, 3.3);
 if (SI_voltage_pin_34 >= 3.7) {
 Serial.print("Problem with ADC on Pin 34; measured voltage=");
 Serial.println(SI_voltage_pin_34);
 ESP_BT.print("Problem with ADC on Pin 34; measured voltage=");
 ESP_BT.println(SI_voltage_pin_34);
 SerialRFD.print("Problem with ADC on Pin 34; measured voltage=");
 SerialRFD.println(SI_voltage_pin_34);
 }


 write_to_file_string += String(SI_voltage_pin_12, 4);
 write_to_file_string += ",";
 write_to_file_string += String(SI_voltage_pin_26, 4);
 write_to_file_string += ",";
 write_to_file_string += String(SI_voltage_pin_34, 4);
 write_to_file_string += ",";


 // Get data from CO2 Sensor
 co2 = get_co2();
 write_to_file_string += String(co2);
 write_to_file_string += ",";


 // Get data from GPS Module
 clearGPS();
 int start_millis = millis();
 while (!GPS.newNMEAreceived()) {
 c = GPS.read();
 status_GPS_module = true;
 if (((millis() - start_millis) >= GPS_timeout) or (millis() < start_millis)) {
 status_GPS_module = false;
 break;
 }
 }
 if (status_GPS_module) {
 GPS.parse(GPS.lastNMEA());
 String last_NMEA = GPS.lastNMEA();
 write_to_file_string += last_NMEA;
 }
 else {
 status_GPS_module = initialize_GPS();
 Serial.println("GPS not working!");
 ESP_BT.println("GPS not working!");
 SerialRFD.println("GPS not working!");
 write_to_file_string += "GPS not working!\n";
 }


 // Write data to internal storage
 if (Status_File_System) {
 write_string_to_file(filename, write_to_file_string);
 }
 else {
 Serial.println("Filesystem is not working!!! Data will not be saved on ESP32!");
 ESP_BT.println("Filesystem is not working!!! Data will not be saved on ESP32!");
 SerialRFD.println("Filesystem is not working!!! Data will not be saved on ESP32!");
 Status_File_System = initialize_littlefs_format_file_system();
 }


 // Print data to all serial interfaces
 SerialRFD.print(write_to_file_string);
 Serial.print(write_to_file_string);
 ESP_BT.print(write_to_file_string);
 delay(1000);
}

# Response correction

## Derivation of the response correction algorithm

When measuring mixing ratios of different gases, it is crucial for the sensors to have the same response time, or, even better, have a 0 s response time, especially in the case of environments with potentially quickly changing concentrations. This ensures that the measured ratio is exactly representing the real ratio of those gases in the atmosphere and not a false ratio caused by different response times. [1, 2, 3] In the past, different techniques have been used to account for those differences, like in [4] or in [2] where the signal of a faster responding sensor was modelled to yield a slower response product. [1] used the same response time correction algorithm, but instead of determining the response time of the sensor in the lab, and then using this response time to calculate the response corrected concentration, they used a method to numerically find the response times to reach the highest cross correlation between the measured signals. This method relies on the necessary cross correlation between the measured analytes and is therefore not applicable when only one sensor is used. However, in the case of volcanic gases, a high cross correlation between the gases can be expected. With the method presented in this work, the concentration can be response corrected, even when only data from one sensor is available or the measured signals are not correlated.

The method used in this paper to correct the measured concentration is based on a mathematical derivation of first order response function of a sensor to a sudden change in concentration (step response). This approach only utilizes the response time (sometimes specified in the datasheet of the sensor) and the measured signal to calculate the response corrected concentration. This concept is computationally very lightweight and fast to implement, which allows for an easy implementation on a microcontroller to directly deliver the response corrected sensor signals.

Equation (S1) describes a step response in a first-order system. [5, 6] In the following considerations this will be the underlying equation that will be used to derive the response time corrected concentration $c_{end}$ of our sensor. A first-order system was used, since only one time constant $\tau$ (the response time) is needed, and for most sensors, this time constant is given in the datasheet. This will later enable us to directly calculate the response corrected concentration, without calibrating or specifying the response time of the sensor on our own. However, an individual calibration to determine the sensor specific response time is advised, as the given response time may strongly differ from the measured response time.

Starting from equation (S1):

| $c\left( t \right)=c_{\text{end}}+\left( c_{\text{start}}-c_{\text{end}} \right)\cdot e^{\frac{-t}{\tau}}$ | **(S1)** |
| --- | --- |

Through a set of calculations, equation (S1) can be rearranged to form equation (S2):

| $c\left( t \right)=\left( c_{\text{end}}-c_{\text{start}} \right)\cdot\left( 1-e^{\frac{-t}{\tau}} \right)+c_{\text{start}}$ | **(S2)** |
| --- | --- |

With $\Delta$ defined as $\Delta=c_{\text{real}}-c_{\text{measured}}$ equation (S2) simplifies to:

| $c\left( t \right)=\left( \Delta\right)\cdot\left( 1-e^{\frac{-t}{\tau}} \right)+c_{\text{start}}$ | **(S3)** |
| --- | --- |

When we now calculate the derivative with respect to time of equation (S3), we get the following expression:

| $\frac{dc}{dt}=\frac{\Delta\cdot e^{\frac{-t}{\tau}}}{\tau}$ | **(S4)** |
| --- | --- |

Now, as we want to calculate the present (the latest), response corrected concentration ($c_{\text{end}}$), we focus on the latest measured data and only look at the latest time step. Therefore, we set $t = 0$. This leads to equation (S5):

| $\frac{dc}{dt}=\frac{\Delta\cdot e^{\frac{-0}{\tau}}}{\tau}=\frac{\Delta\cdot1}{\tau}$ | **(S5)** |
| --- | --- |

When we now multiply by $\tau$ and plug in our definition for $\Delta$, we get the following expression, which describes the present, response corrected concentration $c_{\text{end}}$.

| $c_{\text{end}}=c_{\text{start}}+\Delta=c_{\text{start}}+\frac{dc}{dt}\cdot\tau$ | **(S6)** |
| --- | --- |

Equation (S6) enables us to easily calculate the response corrected concentration. The simplicity of this equation also enables us to incorporate this approach on any modern microcontroller, as one equation directly delivers response corrected concentrations.

## Shifting of measured concentrations

During dataprocessing, the response corrected concentration was time shifted by 14 seconds, so that: $c_{shifted}\left( t \right)=c_{non-shifted}\left( t-14s \right)$. This operation accounts for an additional time lag that the S300 sensor experiences and which can not be corrected by the response time model alone. This value was found numerically after calibration with test gases of known concentration (see Figure 1 and Figure 2). During those calibration runs, also the response time of the S300 sensor was determined to be 40 s.

In Figure 3 data acquired during a lab test, in which the slow responding S300 sensor measured the CO_2_ concentration alongside the fast response smartGAS sensor. The model was applied to calculate response corrected concentrations which were then compared to the fast response concentrations. As can be seen from the lab test, the model provides accurate results, even in a more realistic test.

| 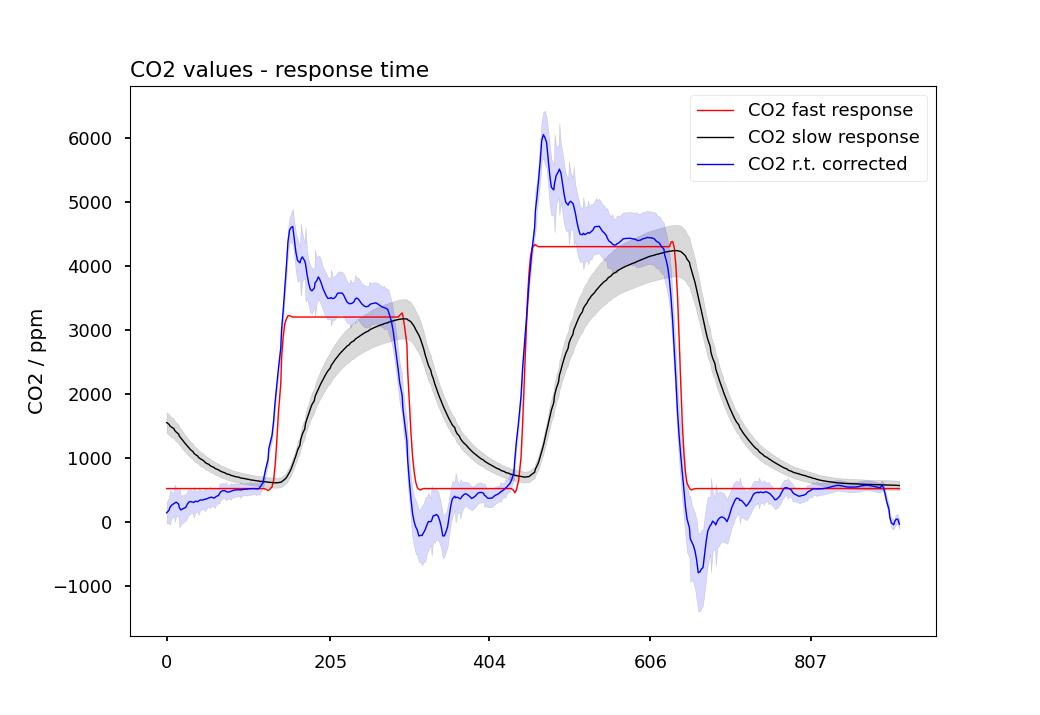  Figure 1: True (red), measured (black) and response time corrected (blue) CO_2_ concentration during calibration with test gases of known concentration. As can be seen, the corrected concentration overshoots in the beginning, indicating that the chosen response time (60 s) was too high. | 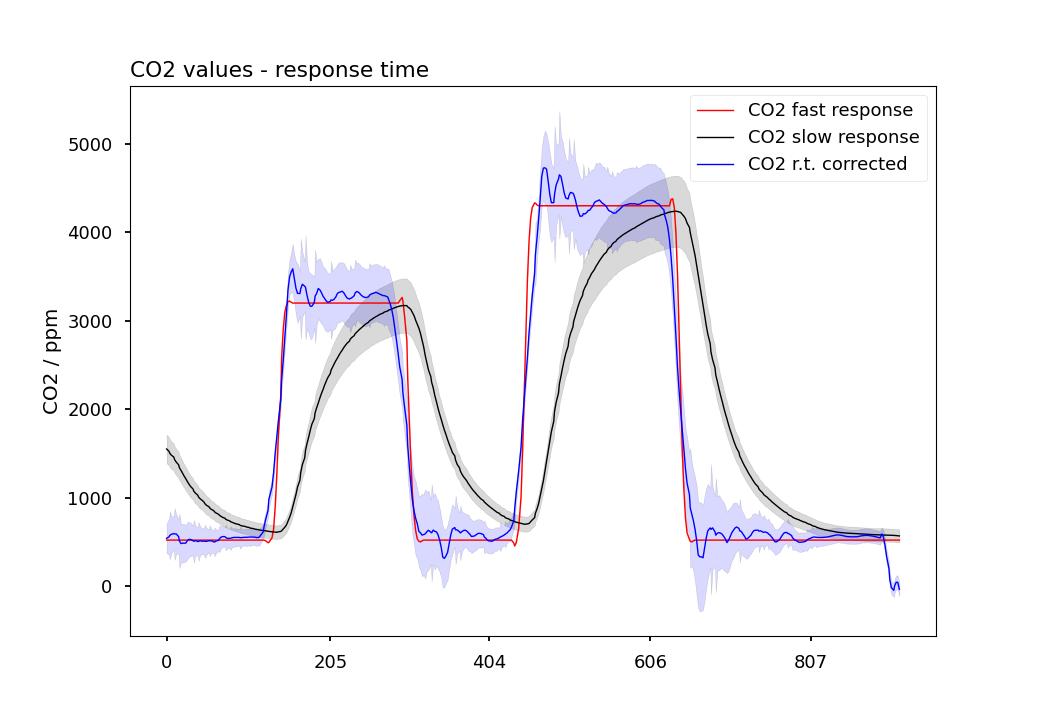  Figure 2: True (red), measured (black) and response time corrected (blue) CO_2_ concentration during calibration with test gases of known concentration. As can be seen, the corrected concentration closely resembles the true concentration, indicating that the chosen response time (40 s) was correct. A response time of 40 s was used to correct all data measured with the S300 sensor. |
| --- | --- |


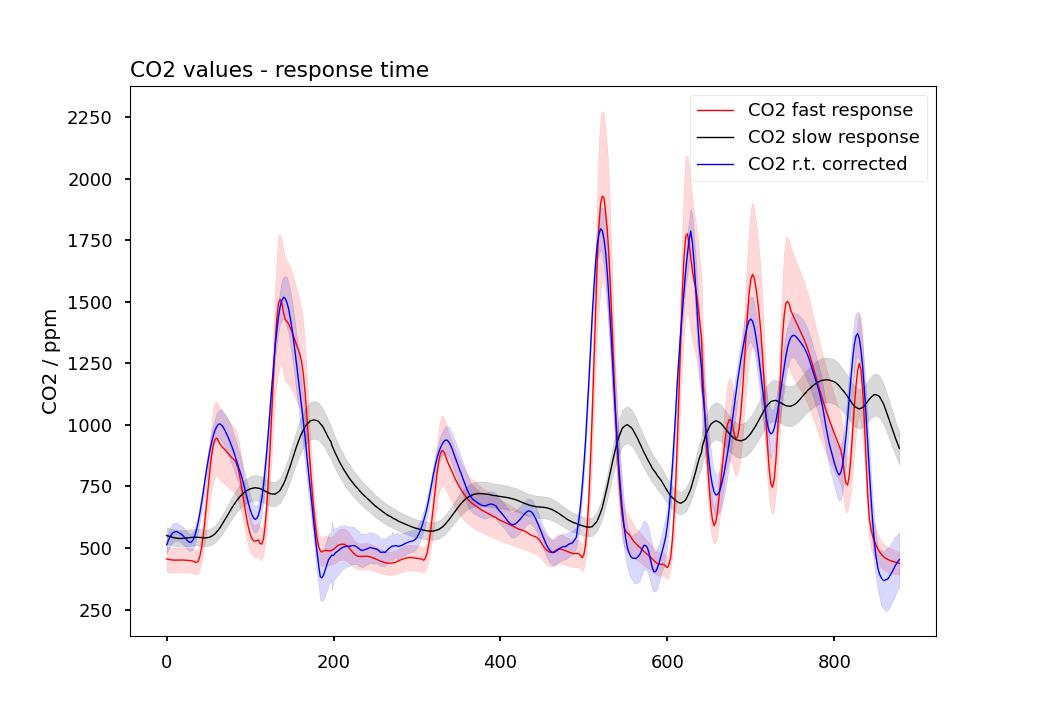


Figure 3: Graph of the fast responding (red), slow responding (black) and response time corrected (blue) CO_2_ concentrations that were measured during a lab test, where different CO_2_ concentration spikes were simulated in a test gas chamber. On the x-axis, the individual time steps are shown.

# Calibration procedure

To calibrate the CO_2_ and SO_2_ sensors, a mixture of CO_2_ and SO_2_ of known concentration was prepared in a gas sampling bag (Tedlar) and then sucked in through the sampling pump and then transferred to the sensors. The calibration was done once before and once after the campaign. To account for drift, the average slope of both calibrations was calculated and used for all concentration measurements. For a more detailed description, see chapter Dataprocessing workflow.

In Table 1 the concentrations used for the calibrations before and after the campaign are shown. The calculated slope is given for both calibrations. For the calibration before the campaign, also the coefficient of determination (R^2^) is given.

Table 1: Data of the CO_2_ and SO_2_ sensor calibration before and after the campaign.

|  | **Before the campaign** | | | | **After the campaign** | | | |
| --- | --- | --- | --- | --- | --- | --- | --- | --- |
|  | CO_2_ [ppm] | measured signal [ppm] | SO_2_ [ppm] | measured signal [V] | CO_2_ [ppm] | measured signal [ppm] | SO_2_ [ppm] | measured signal [V] |
| 1 | 1900 | 2100 | 0.5 | 0.142 | 1010 | 1060 | 5 | 0.877 |
| 2 | 2900 | 3200 | 2 | 0.322 | 1510 | 1608 | 10 | 1.507 |
| 3 | 3900 | 4300 | 4 | 0.569 |  |  |  |  |
| m | 1.100 | | 0.122 | | 1.096 | | 0.126 | |
| R^2^ | 1.000 | | 0.999 | | / | | / | |

As the slope of the calibration did slightly change, the average slope $m_{average}$ was calculated to resemble the actual slope during the campaign.

| $m_{average}=\frac{m_{before+m_{after}}}{2}$ | **(S7)** |
| --- | --- |

The offset $b_{individual}$ was individually determined for each measurement flight by using the averaged measured signal outside the plume $\bar{s_{outside}}$ and calculating the parameter $b_{individual}$ by rearranging the fit equation and subtracting the atmospheric background concentration of the corresponding gas (CO_2_: 420 ppm; SO_2_: 0,0 ppm).

| $b_{individual}=- m_{average}\cdot\bar{s_{outside}}+c_{background}$ | **(S8)** |
| --- | --- |

This results in the following calibration equation, where $s$ is the measured signal.

| $c\left( t \right)=m_{average}\cdot s\left( t \right)+b_{individual}$ | **(S9)** |
| --- | --- |

# Dataprocessing workflow

All, with the in chapter S4 described method, calculated concentrations were smoothed with a Savitzky-Golay filter to reduce noise. [7] Filtering was conducted by applying the *scipy.signal.savgol_filter()* function provided in the *scipy* library written for Python (window = 9, derivation degree = 3).

After smoothing, the response corrected CO_2_ concentrations were calculated by applying the above-described (see chapter S3) mathematical model.

The error of the modelled CO_2_ concentration $\Delta c_{CO_{2}, rtcorrected}\left( t \right)$ was calculated by adding the error of the initial, non response corrected CO_2_ concentration $\Delta c_{CO_{2},initial}\left( t \right)$ to three times the difference quotient of the individual time step (equation (S11)). The error of the initial CO_2_ concentration is calculated according to equation (S10). The final error of the response corrected concentration calculates with equation (S11). This procedure leads to a larger error, when the model needs to correct a large concentration difference, as they occur during short concentration spikes.

| $\Delta c_{CO_{2},initial}\left( t \right)=\left\vert c\left( t \right)\cdot\left( \frac{m_{before}-m_{after}}{m_{average}} \right) \right\vert+3\cdot\sigma$ | **(S10)** |
| --- | --- |
| $\Delta c_{\left( CO_{2},rtcorrected \right)}\left( t \right)=\Delta c_{\left( CO_{2},initial \right)}\left( t \right)+3\cdot\left\vert\frac{\left( c_{CO2,initial}\left( t+\Delta t \right)-c_{CO2,initial}\left( t \right) \right)}{\Delta t} \right\vert$ | **(S11)** |

The error of the individual SO_2_ concentrations $\Delta c_{SO_{2}}\left( t \right)$ is equal to the concentration multiplied with the difference of the slope of the calibration equation measured before and after the campaign $m_{before} - m_{after}$ divided by the average slope $m_{average}$. To this error, three times the standard deviation $\sigma$ [ppm] calculated from blank measurements is added.

| $\Delta c_{SO_{2}}\left( t \right)=\left\vert c\left( t \right)\cdot\left( \frac{m_{before}-m_{after}}{m_{average}} \right) \right\vert+3\cdot\sigma$ | **(S12)** |
| --- | --- |

To calculate the excess-CO_2_/SO_2_ ratio $r$, the background concentration of CO_2_ was subtracted (~420 ppm) from the measured concentration. The error of the ratio $\Delta r$ calculates with the gaussian error propagation to:

| $\Delta r= \sqrt{\left( \frac{\Delta\left[ CO_{2} \right]}{\left[ SO_{2} \right]} \right)^{2}+ \left( \frac{\left[ CO_{2} \right]}{\left[ SO_{2} \right]^{2}}\cdot\Delta\left[ SO_{2} \right] \right)^{2}}$ | **(S13)** |
| --- | --- |

The ratio is then plotted, only if the ratio itself, and the individual errors of the excess-CO_2_ and SO_2_ concentrations exceed their corresponding error.

# Verification of CO_2_ response correction workflow (verification flight)

To verify the response correction algorithm, a verification flight was conducted, in which the sensorsystem of little-RAVEN, alongside a bigger, more sophisticated CO_2_ sensor (smartGAS, actively pumped, fast responding) was attached to a bigger drone and tested in the same plume, as where the other measurement flights were conducted. This was to ensure that the conditions during the verification were exactly the same as during the measurement flights.


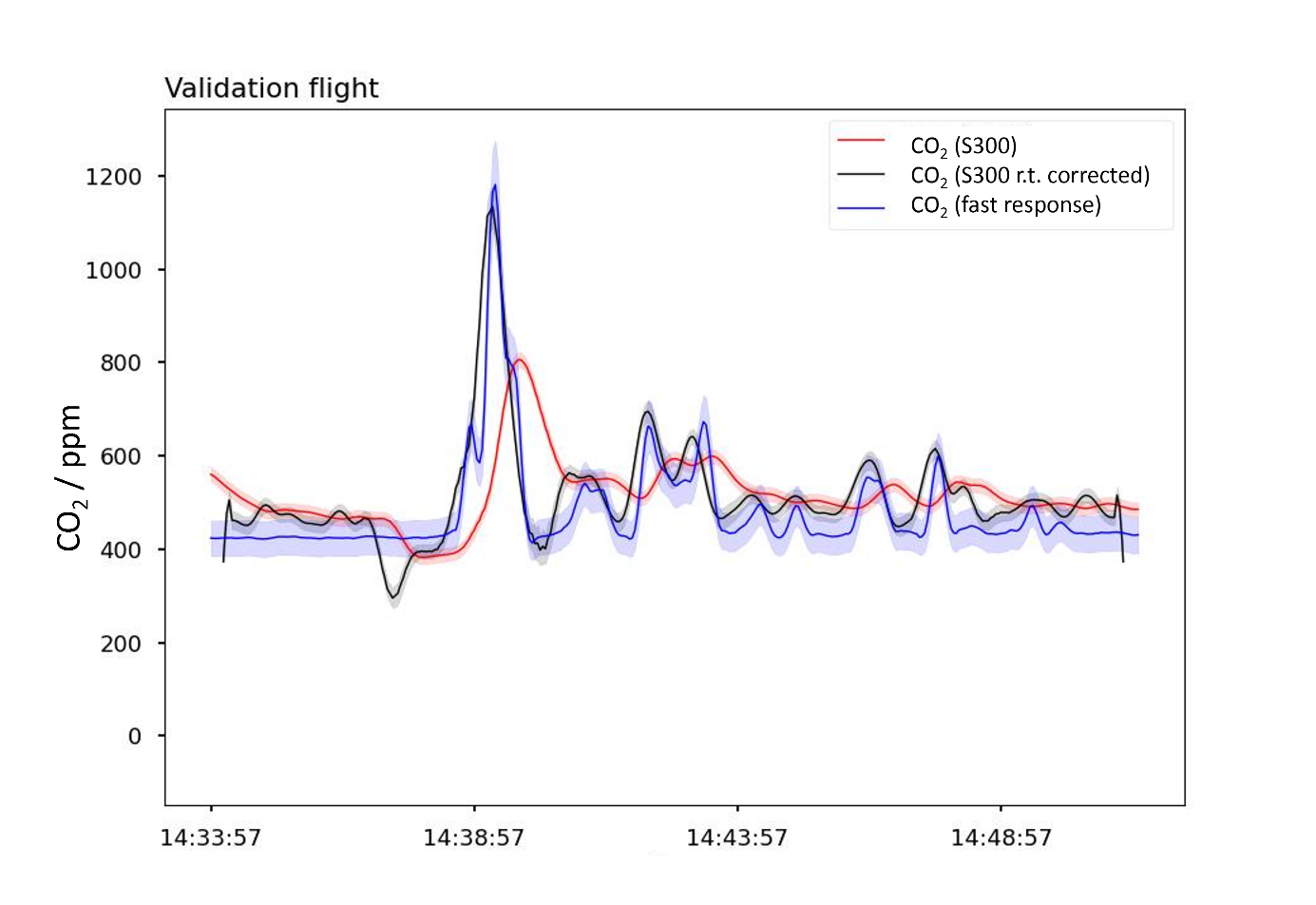


Figure 4: CO_2_ profile as measured during the verification flight (red: CO_2_ concentration as measured with the S300 sensor, black: response corrected CO_2_ concentration; blue: CO_2_ concentration as measured with the fast responding smartGAS sensor). The error was calculated according to the procedure described in the Supplementary Information S5.

In Figure 4 the response corrected CO_2_ concentration is shown in blue. The, with the fast responding sensor measured CO_2_ concentration is shown in red. In black, the uncorrected CO_2_ concentration is shown.

As can be seen in the figure, the graph shows a significant improvement, not only of the relative peak heights, but also of the general concentration profile that was measured during the flight. This leads to more realistic ratios, as the artifacts introduced by differing response times are eliminated.


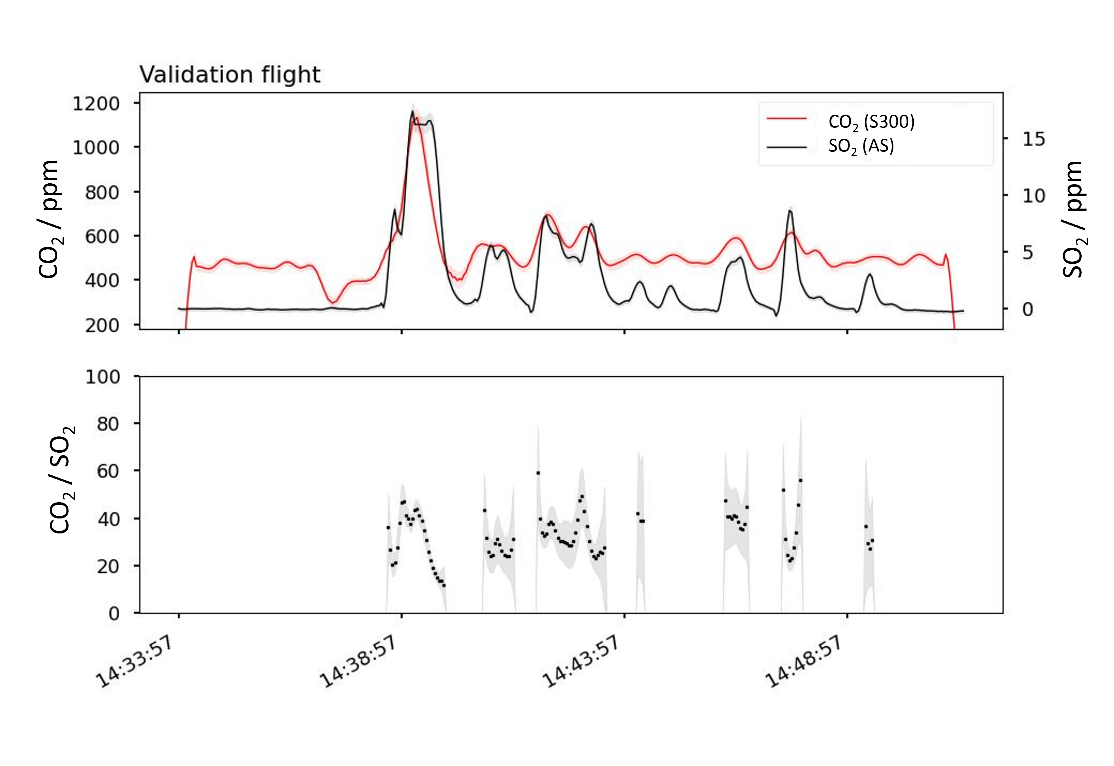


Figure 5: Excess-CO_2_/SO_2_ ratio as measured during the verification flight. The CO_2_ concentration used to calculate this ratio was measured with the S300 sensor and then response corrected. The error was calculated according to the procedure described in the Supplementary Information S5.


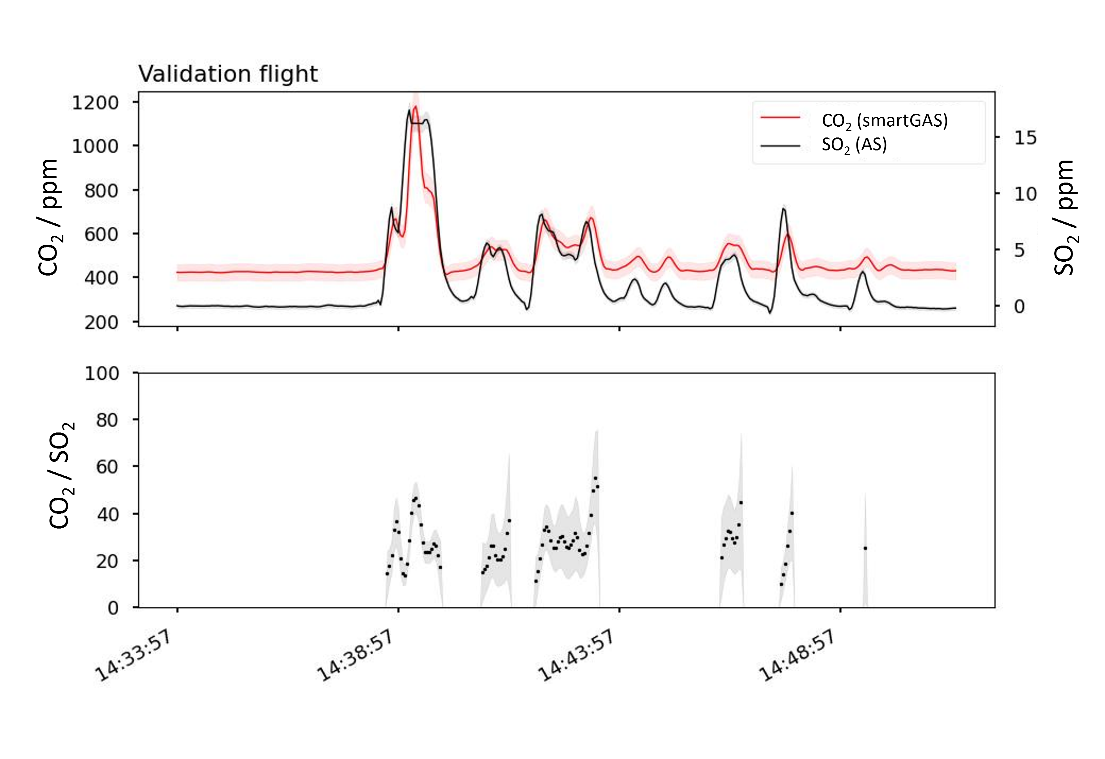


Figure 6: Excess-CO_2_/SO_2_ ratio as measured during the verification flight. The CO_2_ concentration used to calculate this ratio was measured with the fast responding smartGAS sensor. The error was calculated according to the procedure described in the Supplementary Information S5.


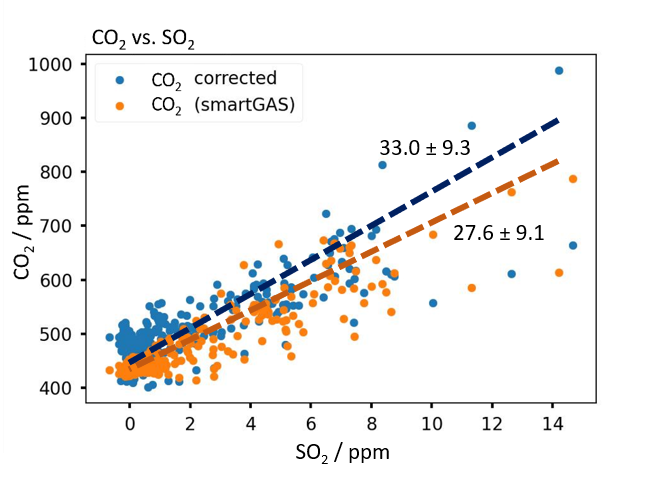


Figure 7: Scatterplot of the CO_2_ concentration as measured with the fast responding smartGAS sensor (orange) and the response corrected S300 sensor (blue) plotted against the SO_2_ concentration.

In Figure 5 and Figure 6, the CO_2_ concentrations measured with the slow but response-corrected sensor (Fig. 5) and the CO_2_ concentrations measured with the fast sensor (Fig. 6) are shown, in each case together with the SO_2_ concentrations measured with the Alphasense SO_2_ sensor. The calculated CO_2_/SO_2_ ratios are also shown in the two figures. The averaged ratios based on the fast response sensor are 27.6 ± 9.1 and 33.0 ± 9.3 for the slow response sensor. This comparison is illustrated in Figure 7. The averaged ratios show that there is no significant difference between the results calculated by the two methods. This demonstrates the applicability of the response correction algorithm.

# Measurement flight 2

The averaged excess-CO_2_/SO_2_ ratio during the second measurement flight was: 32.7 ± 6.8.

The concentration time profile (a)), the excess-CO_2_/SO_2_ ratio (b)) and a scatterplot with corresponding linear regression of the individual concentrations (c)) can be seen in Figure 8.


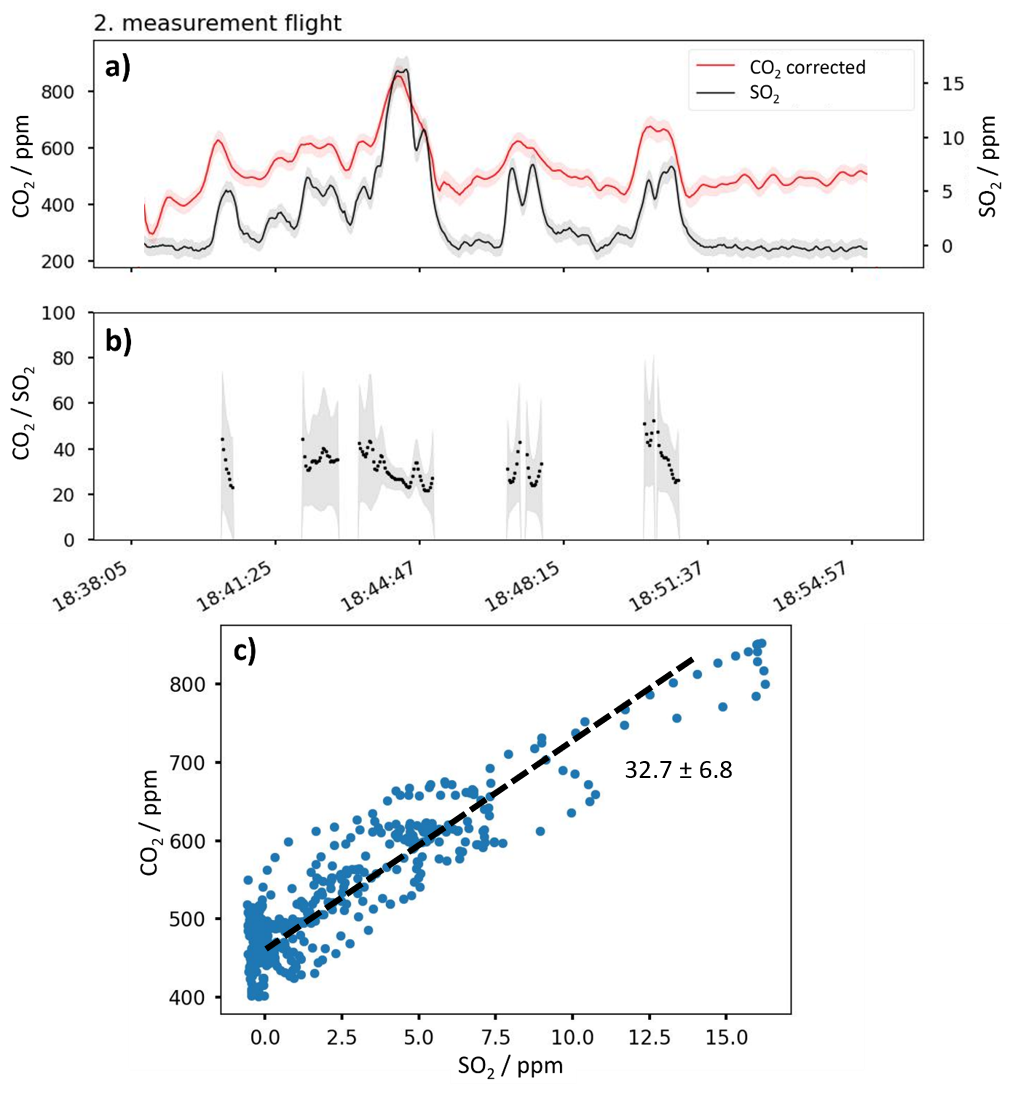


Figure 8: Concentration time profile (a)) and excess-CO_2_/SO_2_ ratio (b)) during measurement flight 2. The error was calculated according to the procedure described in the Supplementary Information S5. c) CO_2_ concentration plotted against the SO_2_ concentration.

# Photoacoustic sensor

During testing, a photoacoustic CO_2_ sensor (Sensiron SCD41) was used. In a lab test, the sensor worked well and delivered stable and correct results. However, once the sensor was put on the drone, the signal became noisy, which became even worse, once the drone became airborne. The two corresponding signals can be seen in Fig. 9 (lab test) and Fig. 10 (drone test).


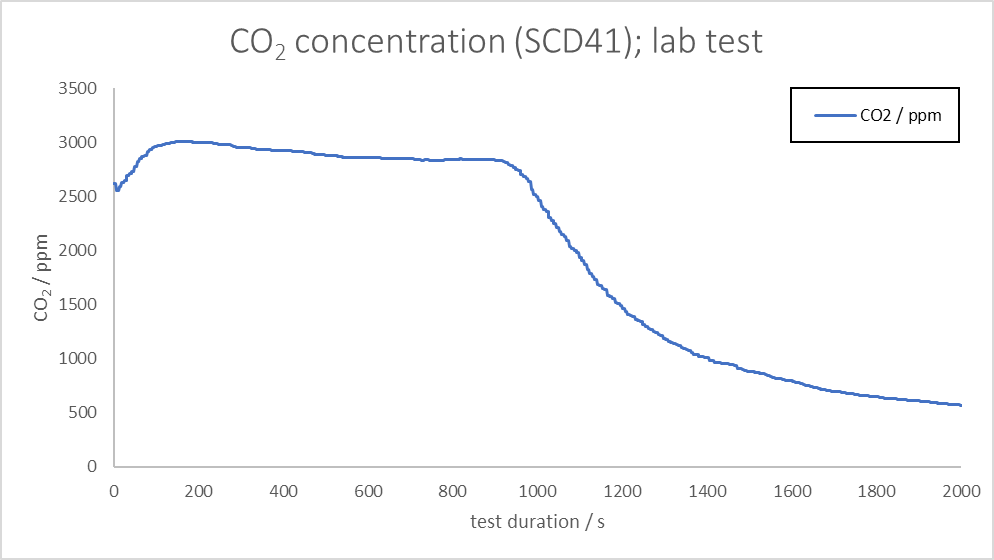


Figure 9: CO_2_ concentration as measured during a lab test. The CO_2_ concentration inside a small, unventilated office was measured. At 1000 s a window was opened.


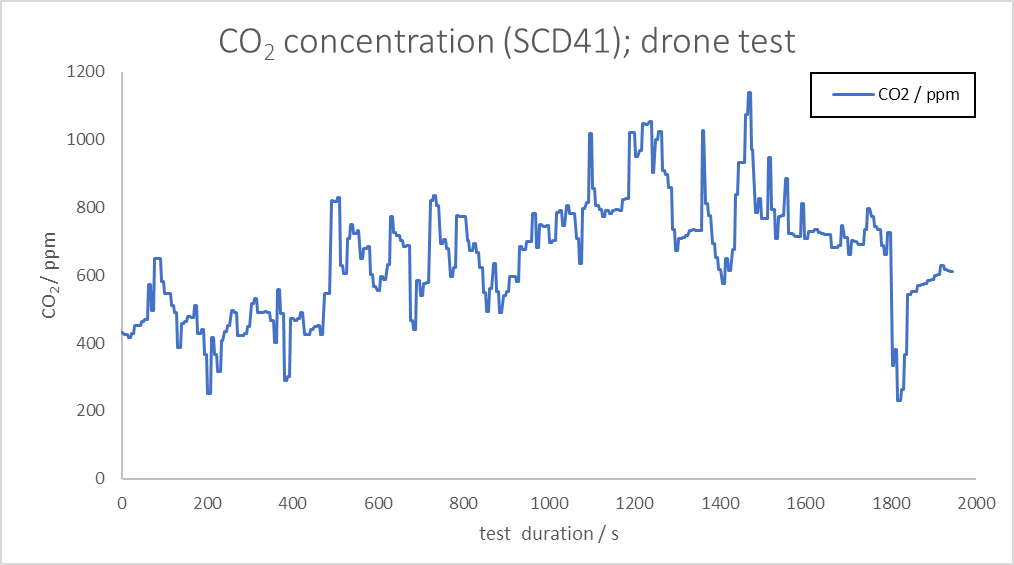


Drone is at the ground

Drone is in the air

Figure 10: CO_2_ concentration as measured during a drone test. The test was performed outside and no CO_2_ emitting sources were present during the test.

It is assumed that RF induced electromagnetic interferences or the spinning drone propellers (and the by those caused pressure fluctuations), or a combination of both, are responsible for the strong noise of the measured concentration during the drone test.

# References

[1] B. Galle et al.: A drone system for long-range and high-altitude volcanic gas plume measurements, *Atmos. Meas. Tech.*, **14**, 4255–4277, (2021), 10.5194/amt-14-4255-2021

[2] J. Rüdiger et al.: Implementation of sensors and sampling techniques on UAV, *Atmos. Meas. Tech.*, **11**, 2441–2457, (2018), 10.5194/amt-11-2441-2018

[3] H. Shinohara: A new technique to estimate volcanic gas composition: plume measurements with a portable multi-sensor system, *Journal of Volcanology and Geothermal Research,* **143**, 319–333, (2005), [10.1016/j.jvolgeores.2004.12.004](https://doi.org/10.1016/j.jvolgeores.2004.12.004)

[4] T.J. Roberts et al.: Electrochemical sensing of volcanic gases, *Chemical Geology,* **332–333**, 74–91, (2012), 10.1016/j.chemgeo.2012.08.027

[5] T.J. Roberts, J.R. Saffell, C. Oppenheimer, T. Lurton: Electrochemical sensors applied to pollution monitoring: Measurement error and gas ratio bias — A volcano plume case study, *Journal of Volcanology and Geothermal Research*, **281** 85 – 96, (2014), 10.1016/j.jvolgeores.2014.02.023

[6] Diana L. Osorio-Arrieta et al.: Reduction of the Measurement Time by the Prediction of the Steady-State Response for Quartz Crystal Microbalance Gas Sensors, *Sensors*, **18**, 2475, (2018), 10.3390/s18082475

[7] A. Savitzky, M. J. E. Golay: Smoothing and Differentiation of Data by Simplified Least Squares Procedures, *Analytical Chemistry*, **36**, 8, 1627-1639, (1964), 10.1021/ac60214a047
